# Lake sturgeon behavioral diversity in the Laurentian great lakes: migratory patterns across populations and habitats

**DOI:** 10.1186/s40462-025-00585-y

**Published:** 2025-10-23

**Authors:** Skye D. Fissette, Charles C. Krueger, Lisa M. O’Connor, Thomas C. Pratt, Daniel A. Isermann, Dan Wilfond, John A. Sweka, Darryl W. Hondorp

**Affiliations:** 1 Department of Fisheries and Wildlife, Michigan State University, East Lansing, MI 48824 USA; 2 Department of Fisheries and Wildlife, Center for Systems Integration and Sustainability, Michigan State University, East Lansing, MI 48824 USA; 3 Ecosystems Sciences Division, Fisheries and Oceans Canada, Fisheries and Oceans Canada, 1219 Queen Street East, Sault Ste. Marie, Ontario, ON P6A 2E5 Canada; 4 U.S. Geological Survey-Wisconsin Cooperative Fishery Research Unit, College of Natural Resources, University of Wisconsin-Stevens Point, 800 Reserve St., Stevens Point, Wisconsin, 54481 USA; 5 Minnesota Department of Natural Resources, Division of Fish and Wildlife, Duluth Field Office, 5351 North Shore Drive, Duluth, MN 55804 USA; 6 U.S. Fish & Wildlife Service, Northeast Fishery Center, 308 Washington Ave, P.O. Box 75, Lamar, PA 16848 USA; 7 Great Lakes Science Center, U.S. Geological Survey, Ann Arbor, MI 48105 USA

**Keywords:** Acoustic telemetry, Divergent migration, GLATOS, Metapopulation, Migratory variability, Partial migration, Migratory variation, Differential migration, Migratory strategy

## Abstract

**Background:**

Characterizing the diversity of migration behaviors from the individual to the population level is essential for understanding how organisms respond to environmental variation and how these responses affect survival and habitat use. Lake sturgeon (*Acipenser fulvescens*) is a species of special concern in the Laurentian Great Lakes that are long-lived and generally classified as intermittent, adfluvial spawners. Observations of lake sturgeon movements at ecologically relevant spatiotemporal scales have shown that migration behavior often varies among individuals within the same population. However, studies on individual populations, particularly when focused only on a part of the life cycle (e.g., often spawning), provide just a partial understanding of the species’ full migratory scope and processes underlying expression of different migratory behaviors. To better understand lake sturgeon migratory diversity, we characterized and compared migratory behaviors of six lake sturgeon populations occupying environments that varied in habitat availability and connectivity in different Laurentian Great Lakes.

**Methods:**

Sequence analysis combined with agglomerative hierarchical clustering and visual inspection of daily location data were used to identify distinct lake sturgeon migratory behaviors present in each population.

**Results:**

Seven distinct migratory behaviors were identified based on differential patterns of lake and river use that encompass spawning and other seasonal periods. Behaviors were categorized as annual spring river, intermittent spring river, intermittent two-step, annual summer river, annual winter river, and annual interlake migrants along with river residents. The presence and frequency of migratory behaviors varied substantially among populations.

**Conclusions:**

Our study demonstrated that migratory diversity is a general feature of lake sturgeon life history that may be partially shaped by habitat availability and connectivity. Given these results, we propose a conceptual model that links habitat availability and connectivity to migratory diversity and predict a positive association between them. This updated framework provides a cohesive basis for understanding lake sturgeon migratory behavior across variable ecological contexts in the Laurentian Great Lakes and will help promote future research to refute or refine the model.

**Supplementary Information:**

The online version contains supplementary material available at 10.1186/s40462-025-00585-y.

## Background

 Migration is a common category of animal movement behavior that is characterized by directed and predictable movements across space and time [1, 2]. Migrating individuals or groups coordinate movements to maximize overlap with critical resources or optimal habitats that enhance fitness outcomes [3–5]. The evolutionary histories of migrating species have been shaped by selection pressures imposed by the distinct environments they inhabit, resulting in a wide range of migration strategies that differ among and within species [6, 7]. Intrapopulation variation in migratory strategies, often termed differential migration (migratory behavior that varies based on individual characteristics such as sex or age; [8–10]), divergent migration [11–13], or migratory portfolios [14–16], results in subgroups that can differ in migration patterns, distances traveled, and migration timing.

Diverse migratory strategies are a common occurrence within fish species [17–19], with examples ranging from protandry (males arriving earlier than females at spawning grounds; [20, 21]), to partial migration (existence of resident and migrating individuals within a population; [22–24]), and divergent migratory patterns within populations that differ in the timing and spatial use of specific habitats [25–27]. Migratory diversity may spread the effects of natural and anthropogenic stressors across population segments, creating a beneficial “portfolio effect” [28, 29] hypothesized to preserve evolutionary potential and promote population stability, resiliency, and persistence in changing environments [30–32]. Therefore, the influence of migratory diversity on population or metapopulation spatial structure [33–35] and the effects of rapidly changing environments on expression of migration behavior [36, 37] are important considerations for fishery management and conservation.

Despite recovery efforts and strong interest in sturgeon (order Acipenseriformes) conservation, much remains to be learned about their movement patterns and spatial distributions, particularly given the centrality of migrations to their life cycle [38]. Sturgeon are long-lived, slow to mature, and spawn intermittently, which makes it difficult to obtain repeat observations of individuals at ecologically relevant timescales (several years) that are needed to characterize patterns in migration behavior. Additionally, migrating sturgeon can travel long distances, and depending on the species, use a variety of river, lake, and ocean habitats [39, 40], which makes them susceptible to environmental disturbances and disruptions to habitat connectivity [41, 42]. Because these life history traits increase vulnerability and species vary in habitats they migrate across, there is a need for species-specific data on migration patterns [43–45].

Lake sturgeon (*Acipenser fulvescens*) is a species of conservation concern across its historical range, which includes the lakes and rivers of the Hudson Bay drainage and the Laurentian Great Lakes (hereafter the Great Lakes), in addition to the upper third of the Mississippi River drainage [46, 47]. They are long-lived (maximum of 154 years; [47]) and slow to achieve first maturity (males, 12–15 years; females 18–27 years; [46]), making them particularly vulnerable to overfishing, which contributed to range-wide declines in their abundance that occurred during the latter part of the 19th and early part of the 20th centuries [48–50]. Widespread habitat loss and fragmentation caused by dams have also contributed to declines and is believed to be the primary factor limiting lake sturgeon recovery [40, 50, 51]. Therefore, understanding variation in migratory behavior and how it shapes the spatial structure of populations is essential for successful management and conservation.

Lake sturgeon spawn intermittently (every 1–3 years for males, 4–9 years for females; [46]) and only in rivers. When ready to spawn, adults migrate to river spawning sites in the spring (typically April-June), reproduce, and then return to lakes or downstream areas of the spawning river [39, 46, 52]. However, a growing recognition exists that alternate migration patterns occur, including protracted use of rivers outside of spawning widows [11, 53, 54]. Some of the most extensive investigations of lake sturgeon spatial ecology in the Great Lakes have occurred in the Huron-Erie Corridor, the unimpeded connecting waterway between lakes Huron and Erie of the Great Lakes that includes the St. Clair River, Lake St. Clair, and Detroit River. Previous studies of lake sturgeon inhabiting this ecosystem [11, 55] identified five migratory behaviors characterized by distinct patterns of river and lake use, which were further categorized into contingents, individuals with the same migratory behavior but that differed in use of specific habitats [11, 13]. For example, individuals residing in Lake St. Clair but spawning in either the Detroit or St. Clair rivers represent distinct contingents [11]. Importantly, these studies revealed previously undocumented complexity in lake sturgeon migratory behavior, raising the question of whether such behaviors occur elsewhere in the Great Lakes.

The limited population, geographic, and temporal scope of most previous studies has hindered a more comprehensive understanding of lake sturgeon migratory behavior in the Great Lakes. Fish movement studies tend to focus on individual populations, which constricts theoretical advancements by promoting an isolated view of behavior potentially biased by population-specific demographics and local geography. For lake sturgeon, a lack of comparative, cross-population behavioral studies precludes an understanding of how the presence and frequency of observed migratory behaviors differs within and across populations and limits insights into how local habitats may shape the expression of specific behaviors. Additionally, observation periods of many previous lake sturgeon movement studies have been too short (≤ 4 years) to fully describe the extent of lake sturgeon migratory diversity and determine the repeatability of individual behaviors. Comparisons of migratory diversity among multiple lake sturgeon populations across long timescales can enhance our understanding of population structures, highlight the importance of key habitats and geographic regions, and inform conservation, management, and restoration efforts [46, 50].

The goal of our study was to characterize the frequency and distribution of lake sturgeon migratory patterns, including movements made for spawning and other functions (e.g., feeding or overwintering), among six distinct lake sturgeon populations occurring in different Great Lakes that vary in habitat availability and connectivity. We used long-term acoustic telemetry (6–10 years) to (1) determine what migratory strategies and population contingents exist and at what frequency for six lake sturgeon populations found in the Huron-Erie Corridor (Lake Huron and Lake Erie), Green Bay (Lake Michigan), the St. Louis River Estuary (Lake Superior), Black Sturgeon River (Lake Superior), eastern Lake Superior, and eastern Lake Erie, (2) provide insights into the possible linkage between observed migration patterns and local habitat availability and connectivity and (3) compare our results with those of previous studies in the Huron-Erie Corridor to evaluate the robustness of previous classifications and identify whether additional migratory strategies and population contingents exist. Sequence analysis [56], combined with agglomerative hierarchical clustering and visual inspection of daily location data, identified distinct migratory behaviors and contingents present in each population, providing new insights into lake sturgeon migratory diversity in the Great Lakes. Based on our results, we also propose a refined conceptual model for lake sturgeon migratory behavior in the Great Lakes that hypothesizes a link between habitat availability and connectivity with migratory diversity.

## Methods

### Study sites

#### Huron-Erie Corridor

**Fig. 1:**
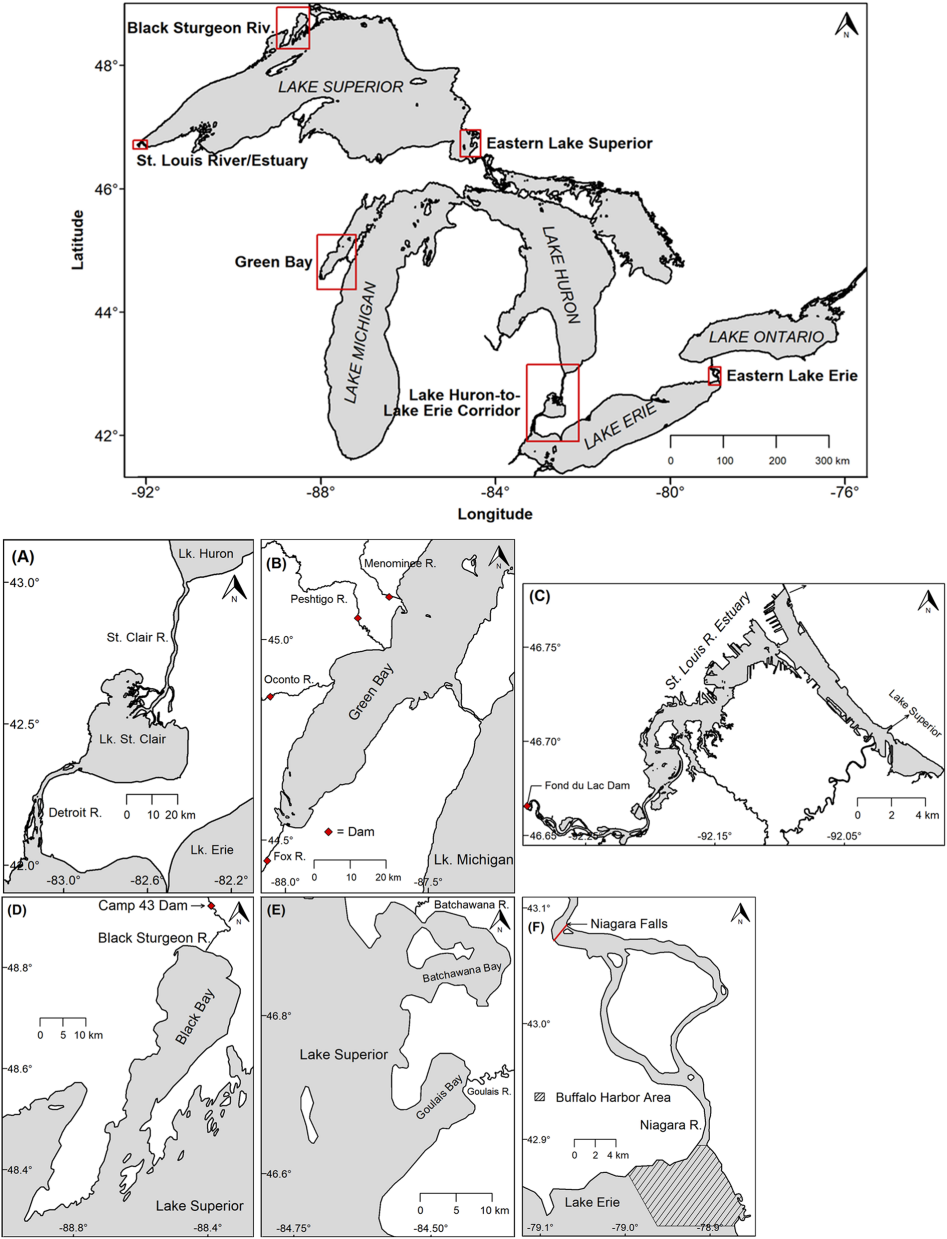
Study site locations for the six lake sturgeon populations investigated in the Laurentian Great Lakes, North America: **(A)** Huron-Erie Corridor, **(B)** Green Bay, **(C)** St. Louis River Estuary, **(D)** Black Sturgeon River, **(E)** eastern Lake Superior, **(F)** eastern Lake Erie. Red boxes denote the boundaries displayed by each map inset and red diamonds indicate dam locations. The cross-hatched section in eastern Lake Erie references the Buffalo Harbor area

The Huron-Erie Corridor is a major connecting channel within the Great Lakes that joins Lake Huron to Lake Erie via the St. Clair River, Lake St. Clair, and Detroit River (Fig. 1). The region spans 135 km and due to a lack of man-made barriers within this connecting waterway, lake sturgeon have complete access to a diverse array of habitats within this region ranging from rivers, a river delta, coastal wetlands, and nearshore and offshore lake environments [57]. Sturgeon movements were also monitored throughout adjacent, large areas of lakes Erie (downstream) and Huron (upstream; Fig. 1A).

#### Green Bay

Green Bay is a shallow (mean depth = 14 m; [58]) embayment of northwestern Lake Michigan that encompasses approximately one third of Lake Michigan’s drainage basin [59–61]. Lake sturgeon had full access to the relatively shallow areas within the bay, as well as broad access at the north end to nearshore and offshore lacustrine habitats of Lake Michigan’s main basin (Fig. 1). Four tributaries emptying into Green Bay support remnant sturgeon populations [60, 62] but access to riverine habitat in each of these systems is limited by dams located close to river mouths (Fig. 1B; Menominee River, 3.7 km upstream from the bay; Fox River, 10.8 km; Oconto River, 23 km; and Peshtigo River 19.2 km; [62]).

#### St. Louis River Estuary

The St. Louis River Estuary is located on the western edge of Lake Superior where it forms the border between states of Minnesota and Wisconsin (Fig. 1). Within this area, ∼ 25 km of unfragmented habitat is available stretching from the Fond du Lac Dam downstream to Lake Superior where the river and lake join via two separate entry channels in Duluth, MN and Superior, WI (Fig. 1C). The uppermost section near the Fond du Lac dam (∼ 10 km) is typical riverine habitat that is relatively shallow and contains some coarse substrate [63] that offers spawning habitat for lake sturgeon (Dan Wilfond, Minnesota Department of Natural Resources, written personal communication; April 30, 2024) along with intact riparian and floodplain zones [64]. This riverine area transitions into the deep and slow lower estuary that has an industrialized shoreline and contains dredged shipping channels surrounded by shallow areas [63–65]. This area may be best classified as a drowned river mouth/estuary ecosystem due to mixing of both lake and riverine habitats [66]. Lake sturgeon also have complete access to nearshore and offshore lake habitats of Lake Superior through the two entry channels.

#### Black Sturgeon River

The Black Sturgeon River is the seventh largest tributary to Lake Superior and empties into Black Bay in the lake’s northwest region (Fig. 1). Lake sturgeon have access to 17 km of river before being blocked by the Camp 43 Dam (Fig. 1D). Black Bay is a relatively shallow, estuary like area that provides productive lake habitat for lake sturgeon feeding [67, 68] and direct access to nearshore and offshore areas of Lake Superior.

#### Eastern Lake Superior (Batchawana and Goulais Rivers)

This region of southeastern Lake Superior is comprised of several relatively shallow and productive bays (Whitefish, Batchawana, and Goulais bays; [69, 70]), two spawning tributaries (Batchawana and Goulais rivers; [70, 71]), and the outlet to the St. Marys River which connects lakes Superior and Huron (Fig. 1E). Additionally, individuals have direct access to nearshore and offshore lake habitats of Lake Superior.

#### Eastern Lake Erie (Buffalo Harbor)

The focal point of this region is located near the headwaters of the Niagara River (Fig. 1F), the outlet at the far eastern end of Lake Erie where a remnant population of lake sturgeon spawn near Buffalo Harbor, NY [72, 73]. Lake sturgeon also had unrestricted access to the remainder of Lake Erie along with the connecting waterways of the Huron-Erie Corridor and its diverse array of habitats located ∼ 390 km away (see *Study Sites: Huron-Erie Corridor*).

### Acoustic Monitoring

As part of a collaborative effort with the Great Lakes Acoustic Telemetry Observation System (GLATOS; https://glatos.glos.us; [74]), movements of acoustic-tagged lake sturgeon from six populations were monitored locally and across the Great Lakes region using an established network of acoustic receivers (69 kHz receivers, Innovasea Systems, Inc., Bedford, Nova Scotia) that are downloaded at least annually. Due to the collaborative nature of the GLATOS acoustic monitoring network, receiver coverage in study areas was temporally and spatially dynamic, but our behavioral classifications generally did not depend on the addition or removal of isolated receiver stations nor minor changes in the location of those stations (e.g., moving a river receiver 1–2 km up or downstream). Documenting every change to receiver distributions during the study period was impractical; however, spatial and temporal variation of receiver deployments within and across regions can be viewed using the GLATOS online receiver mapping tool (https://glatos.glos.us/map), which can be filtered by project codes and/or date. We also provide interactive maps that display locations of all receivers that detected sturgeon from the six populations we investigated (Supplemental Files 1 and 2). The only notable change in receiver coverage that may have affected our results occurred in the Buffalo Harbor and upper Niagara River regions in eastern Lake Erie (crosshatched area in Fig. 1F), where receivers were removed in the fall and not redeployed until the spring, which required us to rely on receiver deployments from other projects in Lake Erie to track movements during the winter months. Therefore, fish movements within the upper Niagara River/Buffalo Harbor area during the winter would not be reliably captured in our data. Because arrays were not positioned close enough to provide fine scale positional data (https://www.innovasea.com/fish-tracking/services/fine-scale-positioning/), a receiver location was assumed to be the fish’s location for each detection.

### Acoustic tagging

The capture and tagging methods used for each population are described in detail in other studies (see Table 1): Huron-Erie Corridor [11, 75], Green Bay [62, 76], St. Louis River Estuary [77], Black Sturgeon River [68], eastern Lake Superior (unpublished but same methods as 68, Lisa O’Connor, Environmental Sciences Division, Fisheries and Oceans Canada, written personal communication; July 18, 2024), and eastern Lake Erie [73]. Capture locations for each population are provided in an interactive map (Supplemental File 3). All studies used 69 kHZ acoustic tags and receivers, and all tags were implanted intracelomically. Sex was determined visually (tagging incision, ultrasound, endoscopy, gamete expression) or molecularly [78].

**Table 1:** Lake sturgeon capture, tagging, and sex determination methods

Population	Capture Method	Tag Model	Sex Determination	*N*	Reference(s)	
Huron-Erie Corridor	Baited Set Lines	V16-6 L	Visual, Molecular	283	[11, 75]	
Green Bay	Dip Net, Electrofishing, Fish Elevator	V16-6 L	Visual	409	[62, 76]	
St. Louis River Estuary	Electrofishing, Dip Nets, Trotline, Gillnet	V16-6X, V16-4X	Visual	163	[77]	
Black Sturgeon River	Gill Net	V16-4X	Molecular	42	[68]	
Eastern Lake Superior	Gill Net	V16-4x, V16TP-4x	Molecular	140	methods same as [68]	
Eastern Lake Erie	Gill Net	V16P-6x, V16TP-6x	Visual	67	[73]	

Overview of capture methods, acoustic tag models, sex determination methods, and the total number of individuals tagged for each population. All tag models were manufactured by Vemco Ltd., now InnovaSea Systems Inc

### Data analysis: Data filtering

Potential false detections created by acoustic tag collisions [79, 80] were removed from each population’s dataset using the *false_detections* function within the R package *glatos* [81], (Huron-Erie Corridor, *N* = 393,224 (1.18%); Green Bay, *N* = 181,522 (1.09%); St. Louis River Estuary, *N* = 62,401 (0.54%); Black Sturgeon River, *N* = 151,671 (2.86%); eastern Lake Superior, *N* = 1,313,025 (2.59%); eastern Lake Erie, *N* = 974,118 (6.49%)). Additionally, any detections that occurred after the 10-year tag life were removed due to concerns that the power of transmissions and their frequency may be compromised (Huron-Erie Corridor, *N* = 10,866; Green Bay, *N* = 4,990).

Multiple filters were used across all populations to remove individuals prior to statistical analyses that were not considered to contribute to our study objectives. Because our goal was to characterize migratory behaviors of adult sturgeon, individuals with total length < 1 m at the time of tagging were presumed to be sexually immature [47] and removed from the data set (St. Louis River Estuary, *N* = 4; eastern Lake Superior, *N* = 31). Additionally, individuals with overall detection histories (time period between first and last detection) shorter than two years were removed (Huron-Erie Corridor, *N* = 16; Green Bay, *N* = 48; St. Louis River Estuary, *N* = 37; Black Sturgeon River, *N* = 2; eastern Lake Superior, *N* = 2; eastern Lake Erie, *N* = 2) and when applicable, individuals with less than a two-year detection history during location history timeframes, see *Daily Location History Timeframes*, (Huron-Erie Corridor, *N* = 9; Green Bay, *N* = 65). A minimum detection history length of two years was chosen because it is the minimum time required to observe repeated annual patterns of previously identified lake sturgeon migratory behaviors [11]. To reduce potential classification errors, detection histories were assessed for evidence of spawning migration(s) by pairing detections that occurred during typical lake sturgeon spawning windows (April – June) to receivers located either in the vicinity of or en route to spawning grounds. This approach aided in identifying and removing individuals who were tagged and then remained in a Great Lake for the entirety of their detection history (Huron-Erie Corridor, *N* = 8; Green Bay, *N* = 1; St. Louis River Estuary, *N* = 24; Black Sturgeon River, *N* = 9; eastern Lake Superior, *N* = 45; eastern Lake Erie, *N* = 4). A population specific filter was also used to remove Green Bay individuals that met the overall two-year detection history filter but had no detections during the location history timeframe while the receiver grid was deployed (see *Daily Location History Timeframes*, Green Bay, *N* = 35).

### Habitat and regional receiver classifications and creating daily location histories

Two location histories were created for each individual [11] that combined temporal (date) and spatial (habitat hydrology or regional) data on a daily timescale. To create daily location histories, habitat hydrology (lake or river) and regional (e.g., Lake Michigan) data were assigned to every receiver that detected a lake sturgeon. Habitat data allowed characterization of general migratory patterns and regional data were used to identify population contingents and further confirm assigned migratory behaviors. The location where a river meets a lake was used to delineate habitat classifications, but a few exceptions were made (Supplemental File 4). To keep the focus on overarching behavioral patterns across populations, a broad classification structure was used for regional data that kept each region as a single, spatial unit. Rivers were never broken up into sections and specific locations within a lake, such geographically defined and named bays, were classified as the lake in which they were located (e.g., Green Bay = Lake Michigan, Fig. 1B). A complete list of all regional assignments is provided (Supplemental File 4), and all habitat and regional assignments for all receivers that detected a lake sturgeon can be viewed on interactive maps (Supplemental Files 1 and 2).

For days when individuals were not detected on the receiver network, we assigned spatial data using last observation carried forward, (LOCF: [82–84]), which assumes an individual remains in the same location until detected in another location. LOCF ensured no missing spatial data within a location history. When individuals were detected in multiple locations on a given day, we assigned the first detected location as the spatial component [55], but LOCF was applied to the last detected location.

### Statistical analyses

#### Daily location history timeframes

Due to changes in receiver coverage across time and different tagging periods across populations, different timeframes were used when creating daily location histories for each population. Our goal was to create the longest possible history while minimizing the potential for spatial classification errors due to limited or changing array coverage. Details describing the daily location history timeframes used for each population are provided in Supplementary File 4.

#### Sequence analyses and identification of migratory behaviors and contingents

All statistical analyses were conducted using R v4.3.2 (https://www.r-project.org/). The R package *TraMineR* [56] was used to generate and analyze habitat and regional sequences created from daily location histories, an approach used previously to analyze migrations in lake sturgeon [55] and walleye [83]. Sequence analysis calculates the edit distance between pairs of sequences, which is the minimum cost of transforming one sequence into another via the sum of assigned costs for two different operations: substitutions or indels. Substitutions occur by swapping one sequence element for another. For example, substituting the element Lake Huron to St. Clair River within a regional sequence. An indel refers to the insertion or deletion of a sequence element that shifts the positions of sequence elements on its right by one position. A custom-defined substitution-cost matrix that reflected geographic distance [55] was used for substitution operations during edit distance calculations between sequences. A cost of zero was assigned when two individuals were in the same habitat/region on a given day, a cost of one when individuals were in adjacent habitats/regions, and a cost of two for non-adjacent habitats/regions. A constant indel cost of 1 was used, which was equal to half our maximum substitution cost and ensured it was never cheaper to perform an insertion or deletion than to perform a substitution. “Left-missing” observations, instances where sequence start dates did not align, were explicitly coded as missing values and given a substitution cost of two. This approach preserved initial temporal alignment for all sequences prior to edit distance calculations by preventing sequences with left missing data from shifting left and therefore aligning with the earliest date present within a population’s dataset. “Right-missing” observations, where behavioral data ended before the final date of the location history time frame, were coded as void elements, removed, and excluded from edit distance calculations. Because some sequences differed in length (e.g., different tagging dates) the optimal matching method with Abbott’s normalization [56] was used and incorporated our custom substitution and specified indel costs. Normalization resulted in dissimilarity measures that did not solely depend on differences in sequence lengths among individuals. Edit distances were calculated for all possible pairwise comparisons of individual sequences to create a matrix of dissimilarity scores.

Migration behaviors were differentiated based on variation in the phenology of adult habitat use and dispersal that considered individual movements among three distinct hydrological units: rivers, nearshore waters of the Great Lakes, and for the Huron-Erie Corridor only, Lake St. Clair. Lake St. Clair was included as a separate unit because it has longer water residence time than rivers but is not typical of nearshore habitats in the Great Lakes. To delineate migratory behaviors, a two-step process was used that first implemented Ward’s agglomerative hierarchical clustering on dissimilarity matrices with the *agnes* function in R package *cluster* [85] followed by a visual inspection of each sequence. The optimal starting number of behavioral groups prior to visual inspection was determined statistically via average silhouette widths (ASW), which range from -1 to 1 and indicate the strength of structuring [86, 87]. ASW values of > 0.5 are considered biologically relevant [87] and this value was used as our benchmark with a max number set to 10 [55]. If multiple behavioral groups had ASW values ≥ 0.5, the most parsimonious solution was chosen, but if no behavioral group values met our benchmark, we chose the one with the highest overall ASW value. Visual inspections included an individual’s entire detection history, including data outside of daily location history timeframes. The initial goal was to use agglomerative hierarchical clustering methods to clearly delineate behavioral patterns in lake sturgeon [11, 55], but statistical separation via ASW values heavily favored grouping based on overall residence times in specific habitats or regions rather than on variation in transition timing between them. Because our goal was to identify migratory behaviors that were dependent on specific transitions between habitats or regions and their timing, we used clustering outputs as an initial guide to group individuals and then visually inspected each sequence to further aid in classifying individuals based on their migratory behavior. Additionally, visual inspection also reduced statistical misclassifications due to (1) the same behavior being observed on time lagged scales, (2) the influence of different sequence lengths on dissimilarity measures, and (3) changes in receiver coverage providing increased resolution of fish movements, thus changing patterns in sequences. Using the behavioral groups identified from habitat sequences, we then visually inspected individuals and assigned them to a migratory behavior.

These analyses were then repeated using regional sequences to (1) confirm the assigned migratory behavior was correct and (2) identify contingents within each population. Confirmation of migratory behavior assignments via regional data was sometimes necessary. For example, the habitat classification “lake” encompassed both the Great Lakes and Lake St. Clair, making it difficult to delineate important migratory transitions between these distinct systems. Regional clustering was conducted for all populations except Black Sturgeon River, where detections were limited to Lake Superior and the river itself, making habitat and regional sequences identical. When a migratory behavior only had one contingent, no ASW values were used to guide subsequent visual inspections. Visual identification of contingents was often necessary due to clustering limitations where small but potentially biologically relevant differences in regional space use were not reliably distinguished (e.g., individuals migrating from the same lake to different spawning rivers). Additionally, an attempt was made to subjectively classify a large subset of individuals (*N* = 100) that were initially excluded from the Green Bay population during data filtering into identified migratory behaviors and contingents based solely on visual inspection. These individuals met all filtering conditions except having a two-year detection history during the location history timeframe.

To determine whether individuals migrating from a lake into a river during the spring for spawning were annual or intermittent migrants, the proportion of years an individual entered the river from March-April (total years detection history/number of spring river trips) was calculated. If the proportion was ≤ 1.5, an individual was classified as annual due to entering the river during spring for a vast majority of its detection history, and if the proportion was > 1.5, an individual was classified as intermittent. Additionally, the percentage of time each statistically classified individual spent in lake(s) was calculated using habitat sequences and then used to calculate the mean lake residency for each migratory behavior within a population.

#### Sex ratios of migration behaviors

To determine if sex ratios were skewed within a migratory behavior and provided evidence for differential migration [8], chi-square goodness of fit tests (α = 0.05) were conducted to compare whether the observed sex ratio within each migratory behavior was different than the sex ratio of classified individuals within the population: Huron-Erie Corridor (1.05:1, M: F, *N* = 227); Green Bay (1.38:1, M: F, *N* = 181). Because molecular sex data was unavailable for the Black Sturgeon River and eastern Lake Superior populations and the overwhelming majority of tagged individuals with a known sex in the St. Louis River Estuary and eastern Lake Erie populations were male, we could only conduct these analyses on the Huron-Erie Corridor (molecular, *N* = 201; visual, *N* = 26) and Green Bay populations (*N* = 181). Individuals with an unknown sex were not used in analyses: Huron-Erie Corridor (*N* = 11), Green Bay (*N* = 4).

## Results

### Data filtering

The number of detections before and after data filtering, along with the number of fish retained for analysis and subsequent behavioral classification post filtering are summarized in Table 2.

**Table 2:** Data filtering results

Population	Pre-Filter Detection Count	Post-Filter Detection Count	Fish Available Post Filtering	Mean ± SD Detections per Fish
Huron-Erie Corridor	33,321,170	32,107,704	245	131,052 ± 169,984
Green Bay	16,659,195	15,801,338	226	69,917 ± 68,488
St. Louis River Estuary	11,531,984	9,550,556	97	98,459 ± 103,437
Black Sturgeon River	5,309,743	3,457,609	18	192,089 ± 139,035
Eastern Lake Superior	50,688,388	8,734,708	28	311,954 ± 247,370
Eastern Lake Erie	15,006,160	8,952,111	56	159,859 ± 169,984
Totals	132,516,640	78,604,026	670	

Summary of detection counts and fish availability for behavioral classification before and after data filtering. Post-filter detection totals include detections outside the daily location history timeframes, as these data were used during visual inspection following cluster analyses. Mean and standard deviation values are rounded to the nearest whole number

### Broad-scale habitat-use patterns of identified migratory behaviors

Statistical clustering and visual inspection of habitat sequences identified three general habitat-use patterns based on the proportion of time individuals resided in lake versus river habitats (Supplemental File 5): “Lake dominant” individuals primarily resided in lakes but were also detected in rivers for short periods of time, whereas “river dominant” individuals occupied rivers exclusively or were only occasionally detected in lakes, and “50:50 lake: river” individuals alternated approximately one-year periods between lake and river habitats. Occurrence of these habitat-use patterns varied across populations (Supplemental File 5). “Lake dominant” individuals were present in all six populations, whereas “river dominant” individuals were present in four of six populations. Only three populations included “50:50 lake: river” individuals (Supplemental File 5).

**Fig. 2:**
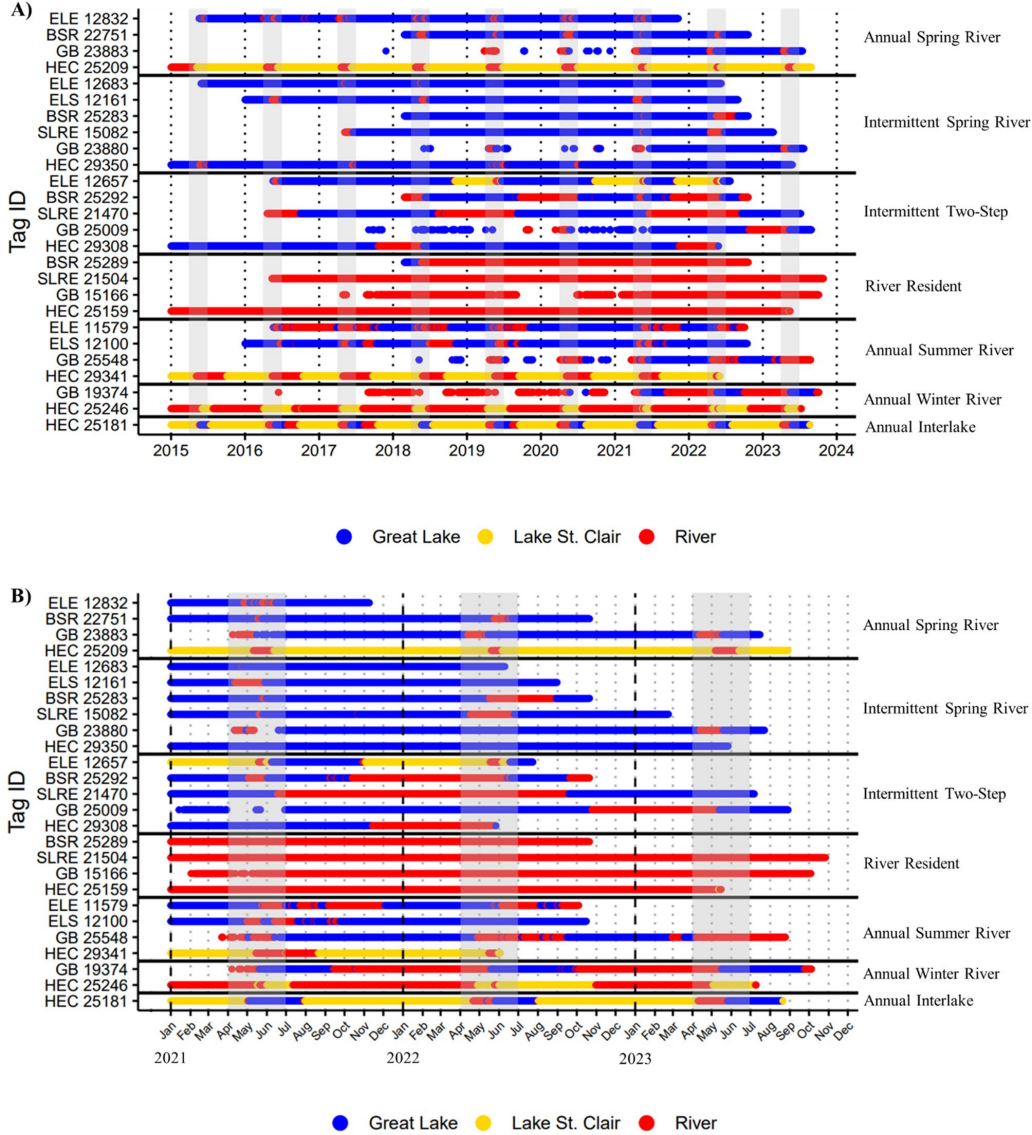
Individual examples of all seven identified lake sturgeon migratory behaviors at **(A)** the full sequence length and **(B)** for a select three-year period to show transition timing between habitats more clearly. A representative sequence was selected for each migratory behavior in populations where they occur, and population names are included with tag IDs and abbreviated: Huron-Erie Corridor (HEC), Green Bay (GB), St. Louis River Estuary (SLRE), Black Sturgeon River (BSR), eastern Lake Superior (ELS), and eastern Lake Erie (ELE). Individuals were classified into migratory behaviors based on agglomerative hierarchical clustering and visual inspection of habitat (lake/river) and regional (e.g., Lk. Michigan) sequence data. Solid horizontal lines delineate migratory behaviors, and gray bars indicate the typical lake sturgeon spawning season in the Laurentian Great Lakes (April-June). Breaks in sequences for GB individuals are due to last observation carried forward not being applied prior to installation of the grid array in that region

With support from clustering algorithms (behavioral group range, 2–4; ASW range, 0.39–0.82, Table 3), subsequent visual inspections of habitat and regional sequences identified seven distinct migratory behaviors embedded within the three general patterns described above in acoustic-tagged lake sturgeon. Migratory behaviors included year-round river residents and six groups of migrants with distinct seasonal patterns of river and lake use (Fig. 2).

**Table 3:** Statistical clustering outputs from habitat sequences

Huron-Erie Corridor	Green Bay	St. Louis River Estuary	Black Sturgeon River	Eastern Lake Superior	Eastern Lake Erie
2 (ASW = 0.81)	4 (ASW = 0.49)	2 (ASW = 0.55)	3 (ASW = 0.39)	2 (ASW = 0.82)	2 (ASW = 0.54)

The optimal starting behavioral group number and the associated average silhouette width values (ASW) used as a guide for identifying migratory behaviors from habitat sequences prior to using visual inspections. ASW values range from -1 to 1 and indicate the strength of group structuring, with ASW values > 0.5 being considered biologically relevant [87]. If multiple behavioral groups had ASW values ≥ 0.5, the most parsimonious solution was chosen, but if no behavioral group values met our benchmark, we chose the one with the highest overall ASW value

### Characterization of migratory behaviors

#### Annual and intermittent spring river migrants (Lake Dominant)

Spring river migrants, termed “lake dominant migrants” by Kessel et al., [11] and “one-step migrants” by Bemis and Kynard [39], made short migrations into a river around spawning season (April -June), followed by an immediate return to a lake after this period (Fig. 2; Supplemental Files 6–11). These individuals were further subdivided based on migration periodicity: annual and intermittent. For instance, individuals BSR 22751 (sex unknown) and HEC 25209 (male) were detected in presumed spawning rivers for ∼ 1–3 weeks every spring during their detection history, whereas GB 23880 (male) was detected in the river near spawning season every other year (Fig. 2). Across all populations both annual and intermittent individuals spent a majority of their time in a lake (annual range, 86–95%; intermittent range, 92–99%; Table 4). The reported range of lake residency here and for subsequent migratory behaviors reflects the mean lower and upper bounds among populations (Table 4).

**Table 4:** Lake residency for each migratory behavior

Migratory Behavior	Huron-Erie Corridor	Green Bay	St. Louis River Estuary	Black Sturgeon River	Eastern Lake Superior	Eastern Lake Erie
Annual Spring River	86 ± 8 (30)	91 ± 6 (8)		91 ± 4 (3)	98 ± 2 (24)	95 ± 5 (29)
Intermittent Spring River	94 ± 6 (80)	96 ± 3 (17)	94 ± 1 (5)	92 ± 9 (3)		99 ± 1 (22)
Intermittent Two-Step (Lake Dominant)	88 ± 6 (11)	*	75 ± 11 (45)	77 ± 12 (8)		95 (1)
Intermittent Two-Step (50:50)	42 ± 9 (3)	*		52 ± 5 (3)		
River Resident	5 ± 9 (33)	5 ± 4 (3)	1 ± 2 (44)	5 (1)		
Annual Summer River	66 ± 14 (42)	68 ± 9 (20)			89 ± 4 (4)	69 ± 10 (3)
Annual Winter River	23 ± 12 (17)	29 ± 13 (18)				
Annual Interlake	71 ± 12 (22)					

Percentage of time (Mean ± SD) each migratory behavior within a population spent in lakes. Percentages were calculated from habitat sequences used in sequence analyses, and the number in parentheses represents the sample size. The percentage of lake residency could not be calculated for Green Bay individuals undergoing intermittent two-step migrations because the short analysis timeframe often did not allow the full migratory pattern to be repeated. This resulted in unreliable calculations that did not adequately capture the proportion of time individuals spent between lake and river habitats, and these instances are denoted by an asterisk. Empty cells denote the absence of a migratory behavior within a population

#### Intermittent two-step migrants (lake dominant or 50:50 lake: river residence)

Intermittent two-step migrations were initiated in the fall when an individual moved into the spawning river, overwintered near the spawning location, and then made a short upstream migration to spawning grounds in spring followed by a return to a lake or downstream section the spawning river (Fig. 2; Supplemental Figs. 6–9, 11) [39, 46, 88]. Habitat classifications were not subdivided to the level of “near spawning grounds” or “spawning grounds” [39], so the fine-scale habitat transitions associated with this behavior could not be displayed graphically. The closest our plots come to capturing these details is for individuals that overwintered in Lake St. Clair, which could be considered “near spawning grounds” and the short river migration after overwintering as movement to “spawning grounds” (e.g., ELE 12657, Fig. 2). This behavior was either lake dominant within a population (range, 75–95%; Table 4) or approximately 50:50 residency between lake and river habitats (range, 42–52%; Table 4). Lake residency could not be calculated for Green Bay individuals because the short analysis timeframe often did not allow the full migratory pattern to be repeated. This resulted in unreliable calculations that did not adequately capture the proportion of time individuals spent between lake and river habitats.

#### River residents (river dominant)

River residents [11, 55, 89] spent nearly the entire observation period residing in one river with minimal movements to a lake (range, 1–5%; Table 4) or neighboring rivers (Fig. 2; Supplemental Files 6–9).

#### Annual summer river (lake dominant)

Annual summer river migrants, termed “seasonal river (summer) – lake (winter)” by Kessel et al., [11], were characterized by movements from a lake to a river during the spring (∼ May) where river presence extended beyond the spawning season into the summer/early fall (∼ Sept.) and was followed by a return to the lake for overwintering (Fig. 2; Supplemental Files 6, 7, 10, and 11). Across all populations, this behavioral group spent most of their time in a lake (range, 66–89%; Table 4), and most individuals likely spawn shortly after entering the river prior to their summer residence.

#### Annual winter river (river dominant)

Annual winter river migrants, termed “seasonal lake (summer) – river (winter) – lake (winter)” by Kessel et al., [11], displayed the inverse pattern of annual summer river migrants (Fig. 2, GB 19374). These individuals moved from a lake to a river in late summer or early fall (∼ Jul.-Oct.) for overwintering, followed by a return to the lake in the spring (∼ Apr-May) where they reside during the spring and summer months (Fig. 2; Supplemental Files 6 and 7). This behavioral pattern often mirrors that of intermittent two-step migrants but occurs annually instead of intermittently. This behavioral group spent less time in lakes than rivers (range, 23–29%; Table 4), and individuals likely spawn shortly before returning to the lake for the spring/summer.

#### Annual interlake (lake dominant)

Annual interlake migrants, termed “lake skippers” by Kessel et al., [11], made movements between multiple lakes via connecting river channels and were only present in the Huron-Erie Corridor population. They overwintered in Lake St. Clair (∼ Sept. - Apr.), then moved up the St. Clair River or down the Detroit Rivers in the spring (∼ Apr. - May), to either Lake Huron or Erie where they spent the summer months (∼ Jun. - Aug.) before returning to Lake St. Clair in the fall to overwinter (Fig. 2; Supplemental File 6). This behavioral group spent a majority of time in a lake (mean = 71%; Table 4), and individuals likely spawn in the St. Clair or Detroit rivers during their transits between Lake St. Clair and lakes Huron or Erie.

### Patterns of migratory behavior varied across populations

A total of 618 individuals were classified into migratory behaviors (Table 5): Huron-Erie Corridor (*N* = 238), Green Bay (*N* = 185), St. Louis River Estuary (*N* = 94), Black Sturgeon River (*N* = 18), eastern Lake Superior (*N* = 28), and eastern Lake Erie (*N* = 55). The occurrence and frequency of each behavior varied across populations, but all populations had more than one migratory behavior present (range 2–7; Table 5). The two typical lake sturgeon migratory behaviors most commonly referenced in the literature, spring river (annual and intermittent) and intermittent two-step migrants [39, 46], were the most frequently observed (range 4–6 populations), comprised a majority of classified individuals in every population (range 53–95%), and represented 64% of all 618 classified individuals (Table 5). River residents (*N* = 4 populations; 14% of classified individuals) and annual summer river migrants (*N* = 4 populations; 13% of classified individuals) were also observed relatively frequently, but annual interlake and annual winter river migrants were far less common, appearing in only one and two populations and representing 6% and 4% of classified individuals, respectively (Table 5). Several individuals could not be classified due to sequences not conforming with the identified patterns of migratory behavior (Huron-Erie Corridor, *N* = 7; Green Bay statistical clustering, *N* = 9; Green Bay visual classification only, *N* = 32; St. Louis River Estuary, *N =* 3; eastern Lake Erie, *N* = 1; Supplemental File 12).

**Table 5:** Variation in migratory behaviors among populations

Migratory Behavior	Huron-Erie Corridor	Green Bay	St. Louis River Estuary	Black Sturgeon River	Eastern Lake Superior	Eastern Lake Erie	Total Frequency
Annual Spring River	30 (13%)	17 (9%)	0	3 (17%)	0	29 (53%)	79 (13%)
Intermittent Spring River	80 (34%)	35 (19%)	5 (5%)	3 (17%)	24 (86%)	22 (40%)	169 (27%)
Intermittent Two-Step	14 (6%)	74 (40%)	45 (48%)	11 (61%)	0	1 (2%)	145 (23%)
River Resident	33 (14%)	8 (4%)	44 (47%)	1 (6%)	0	0	86 (14%)
Annual Summer River	42 (18%)	30 (16%)	0	0	4 (14%)	3 (6%)	79 (13%)
Annual Winter River	17 (7%)	21 (11%)	0	0	0	0	38 (6%)
Annual Interlake	22 (9%)	0	0	0	0	0	22 (4)
Total Classified	238	185	94	18	28	55	618

Cross population comparisons of the occurrence and frequency of identified lake sturgeon migratory behaviors. Totals for Green Bay include subjectively classified fish that did not meet clustering criteria and were assigned via visual inspection only (*N* = 68). Percentages were rounded to the nearest whole number

### Variations within the broader patterns of migratory behavior

**Fig. 3:**
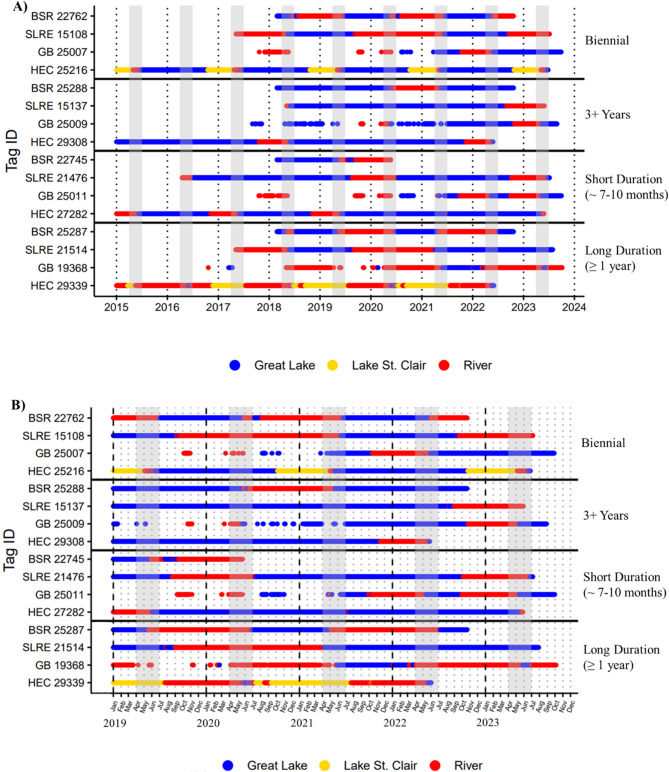
Individual examples showing the variation in migration periodicity, duration, and number of habitat transitions for intermittent two step migrants at **(A)** full sequence length and **(B)** a select 5-year period to show patterns in migration timing more clearly. A representative sequence was selected for each variation observed within intermittent two-step migrations, and population names are included with tag IDs and abbreviated: Huron-Erie Corridor (HEC), Green Bay (GB), St. Louis River Estuary (SLRE), Black Sturgeon River (BSR). A delineation showing the number of habitat transitions is not displayed because individual HEC 25,216 is the only individual plotted undergoing two habitat transitions, whereas all other individuals undergo one habitat transition. Solid horizontal lines delineate variations observed within intermittent two-step migrations, and gray bars indicate the typical lake sturgeon spawning season in the Laurentian Great Lakes (April-June). Breaks in sequences for Green Bay individuals are due to last observation carried forward not being applied prior to installation of the grid array in that region

Long-term, ecosystem-scale tracking of individual movements captured repeated, complete migratory cycles that revealed additional variation within broader migratory patterns. For example, we discovered the existence of intermittent two-step behaviors that differed in migration periodicity (biennial; 3 + years), river residency period (short ∼ 7–10 months; long ≥ 1 year), and number of habitat transitions (one or two; Fig. 3). One habitat transition occurred when individuals migrated directly from a Great Lake to their presumed spawning river in the fall, and two transitions occurred when individuals migrated in the fall from a Great Lake through a river to Lake St. Clair for overwintering, followed by a migration to a spawning river in the spring. The identified habitat-use pattern of 50:50 river: lake residency was a direct result of long duration, biennial intermittent two-step migrants that alternate approximately one year periods between the river and lake (HEC 29339, GB 19368, BSR 25287 in Fig. 3; Supplemental Files 6, 7, and 9). This 50:50 river: lake pattern was most prominent in the Green Bay population, where a large proportion of individuals displayed this behavior with movements between Green Bay/Lake Michigan and the Menominee River (Supplemental File 7).

**Fig. 4:**
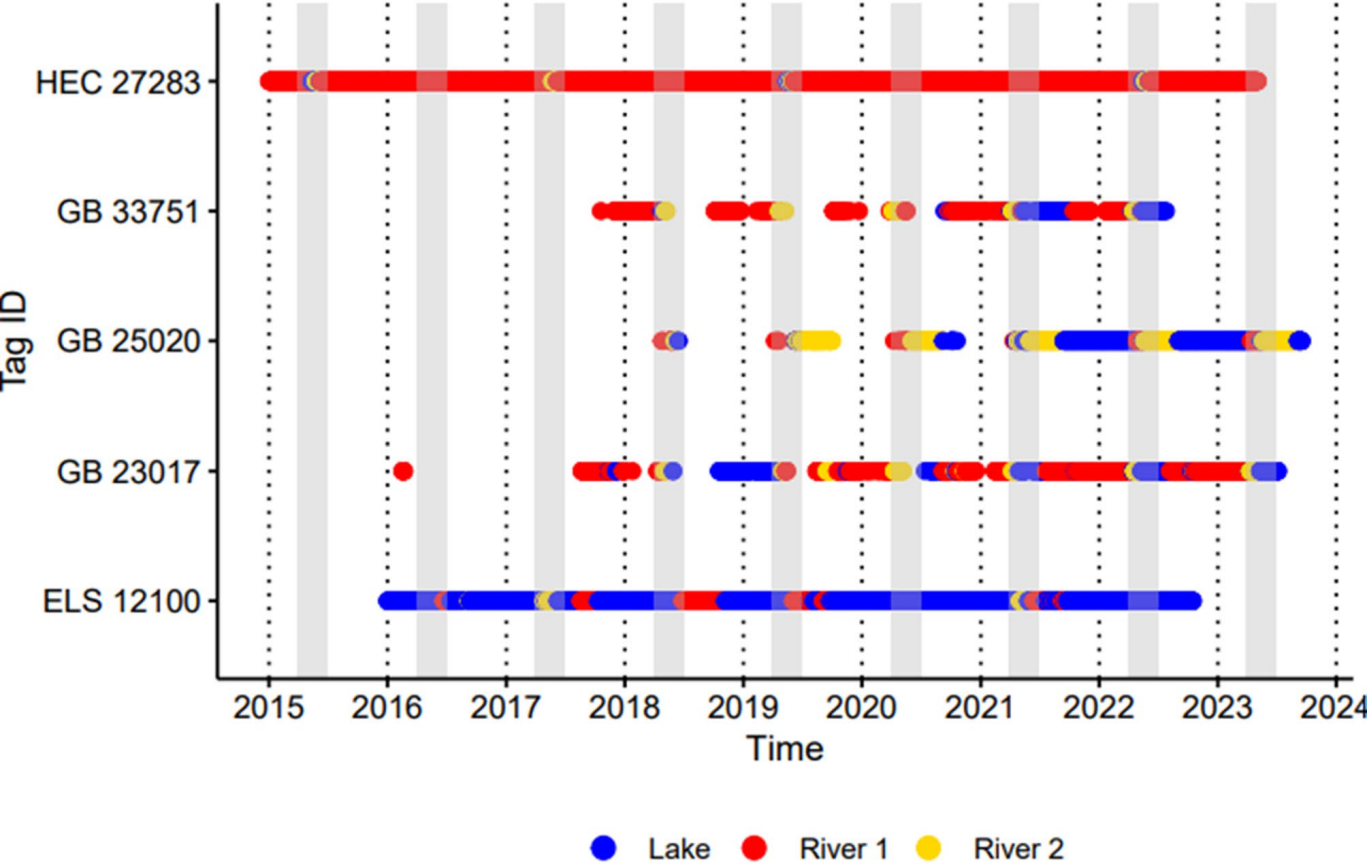
Individual examples of multi-river migrations that were repeated across years and consistent in seasonal timing. Gray bars indicate the typical lake sturgeon spawning season in the Laurentian Great Lakes (April-June) and breaks in sequences for Green Bay individuals are due to last observation carried forward not being applied prior to installation of the grid array in that 1region. Population names are included with tag IDs and abbreviated: Huron-Erie Corridor (HEC), Green Bay (GB), and eastern Lake Superior (ELS)

Some individuals were observed undergoing migrations into multiple rivers that were consistent in timing and repeatable across years but varied in the duration of time spent in each river (Fig. 4). For instance, GB 23,017 (Fig. 4) followed a predictable annual migration pattern, traveling to the Menominee River to overwinter before moving to the Peshtigo River during spawning season. On the other hand, HEC 27,283 (Fig. 4) resided primarily in the Detroit River but may have undergone spawning migrations to the St. Clair River every other year. Most annual summer river migrants were detected only in a single river during both the spawning and summer periods (Supplemental Files 6, 7, 10, 11), but all eastern Lake Superior and a few Green Bay individuals were detected in one river during the spawning period and in a different river during the summer period (e.g., ELS 12100 and GB 25020; Fig. 4; Supplemental Files 7 and 10).

Several individuals were observed making long-distance migrations exceeding the range observed for most other individuals in their population. Two eastern Lake Erie individuals crossed the entire expanse of Lake Erie westward to reach the Huron-Erie Corridor region (ELE 12657 Fig. 2; ELE 11575 Supplemental File 11), some St. Louis River Estuary individuals were detected north in the Black Sturgeon River and east in the Keweenaw Waterway (Supplemental File 8), one eastern Lake Superior individual traversed westward across Lake Superior to presumably spawn in the St. Louis River Estuary (Supplemental File 10), and some Green Bay individuals were detected in Grand Traverse Bay on the east side of Lake Michigan and to the north in Lake Huron (Supplemental File 7).

### Migratory behavior is consistent within individuals

For the Huron-Erie Corridor population, the consistency of our behavioral classifications of individuals previously assigned by Kessel et al., [11] corroborate their suggestion that migratory behaviors were consistent and individuals do not switch behavior over time. Of the 215 individuals shared between the Kessel et al. study and our study, we only reclassified 34 fish (16%), including 14 fish simply because longer detection histories were available that clearly revealed behavioral trends (e.g., intermittent two-step migrants) and 20 fish because of increased receiver coverage providing greater resolution of individual behavior. These instances were outside Kessel et al.’s [11] control and provide clear evidence that individual level migratory behavior is highly repeatable over time in lake sturgeon.

### Contingent structures varied across populations

The number of identified contingents varied across populations with the total number of contingents being positively correlated with the number of identified migratory behaviors and number of regions used by individuals: Huron-Erie Corridor (*N* = 27), Green Bay (*N* = 21), St. Louis River Estuary (*N* = 3), Black Sturgeon River (*N* = 4), eastern Lake Superior (*N* = 3), and eastern Lake Erie (*N* = 4; Table 6). The starting contingent number prior to visual inspection of regional sequences varied by migratory behavior and population (Table 7): Huron-Erie Corridor, contingent range ( [2] – [3]), ASW range (0.36–0.92); Green Bay, contingent range [2–8], ASW range (0.21–0.81); eastern Lake Superior, 2 contingents, ASW (0.82). Visual inspection of contingents identified via clustering often revealed a more complex contingent structure than statistical methods could capture, particularly within the Huron-Erie Corridor and Green Bay populations. These two populations showed greater spatial variability and more complex patterns of regional use compared to the other four populations. Plots for each population showing residence within and transitions between different regions for each migratory behavior and contingent are provided (Supplemental Files 6–11).

**Table 6:** Population contingent structures

Migratory Behavior	Huron-Erie Corridor	Green Bay	St. Louis River Estuary	Black Sturgeon River	Eastern Lake Superior	Eastern Lake Erie
Annual Spring River	LSC → DR (7) LSC → SCR (15) LH → SCR (5) LE → DR (3)	LM → MR. (4) LM → PR. (8) LM → OR. (3) LM → FR (1) LM → Multiple Rs. (1)		LS → BSR (3)		LE → NR (29)
Intermittent Spring River	LSC → DR (11) LSC → SCR (43) LH → SCR (25) LE → SCR (1)	LM → MR. (9) LM → PR. (14) LM → OR (8) LM → FR (2) LM → Multiple Rs. (2)	LS → SLRE (5)	LS → BSR (3)	LS → GR (23) LS → SLRE (1)	LE → NR (22)
Intermittent Two-Step	LSC → SCR (1) LSC → DR (1) LH → SCR (4) LH → LSC → DR (1) LH → LSC → SCR (5) LE → DR (1) LE → LSC → DR (1)	LM → MR (65) LM → PR (1) LM → OR (1) LM → FR (7)	LS → SLRE (45)	LS → BSR (11)		LE → LSC → SCR (1)
River Resident	DR (16) SCR (17)	MR (8)	SLRE (44)	BSR (1)		
Annual Summer River	LSC → SCR (37) LSC → DR (4) LE→ DR (1)	LM → MR. (27) LM → OR → MR (3)			LS → SMR (4)	LE → NR (3)
Annual Winter River	LSC → DR (2) LSC → SCR (4) LSC → LE → DR (1) LE → DR (10)	LM → MR (18) LM → FR (1) LM → FR → OR (1) LM → MR → PR. (1)				
Annual Interlake	LSC → DR → LE (18) LSC → SCR → DR → LE (1) LSC → SCR → LH (3)					
Total Contingents	27	21	3	4	3	4

The contingent structure for each population and regional movement patterns for each contingent. Numbers in parentheses represent the number of individuals within a given contingent, and all regional locations are abbreviated: Lake St. Clair (LSC), Lake. Huron (LH), Lake Erie (LE), Lake Michigan (LM), Lake Superior (LS), Detroit River (DR), St. Clair River (SCR), Menominee River (MR), Peshtigo River (PR), Oconto River (OR), Fox River (FR), St. Louis River Estuary (SLRE), Black Sturgeon River (BSR), Goulais River (GR), St. Marys River (SMR), Niagara River (NR). Empty cells denote to the absence of a migratory behavior within a population

**Table 7:** Statistical clustering outputs from regional sequences

Migratory Behavior	Huron-Erie Corridor	Green Bay	St. Louis River Estuary	Black Sturgeon River	Eastern Lake Superior	Eastern Lake Erie
Spring River Migrants	2 (ASW = 0.81)	2 (ASW = 0.66)	0	0	2 (ASW = 0.82)	0
Intermittent Two-Step	2 (ASW = 0.36)	2 (ASW = 0.60)	0	0		0
River Resident	2 (ASW = 0.92)	0	0	0		
Annual Summer River	2 (ASW = 0.60)	8 (ASW = 0.21)			0	0
Annual Winter River	2 (ASW = 0.84)	2 (ASW = 0.81)				
Annual Interlake	3 (ASW = 0.84)					

The optimal contingent number and the associated average silhouette width values (ASW) used as a guide for determining contingent structures from regional sequences prior to visual inspections. Empty cells denote to the absence of a migratory behavior within a population, and a 0 indicates no ASW value was used for contingent identification. ASW values range from -1 to 1 and indicate the strength of structuring, with ASW values > 0.5 being considered biologically relevant [87]. If multiple contingent options had ASW values ≥ 0.5, the most parsimonious solution was chosen, but if no ASW values met our benchmark, we chose the contingent with the highest overall ASW value

### Sex ratios varied among migratory behaviors

**Fig. 5:**
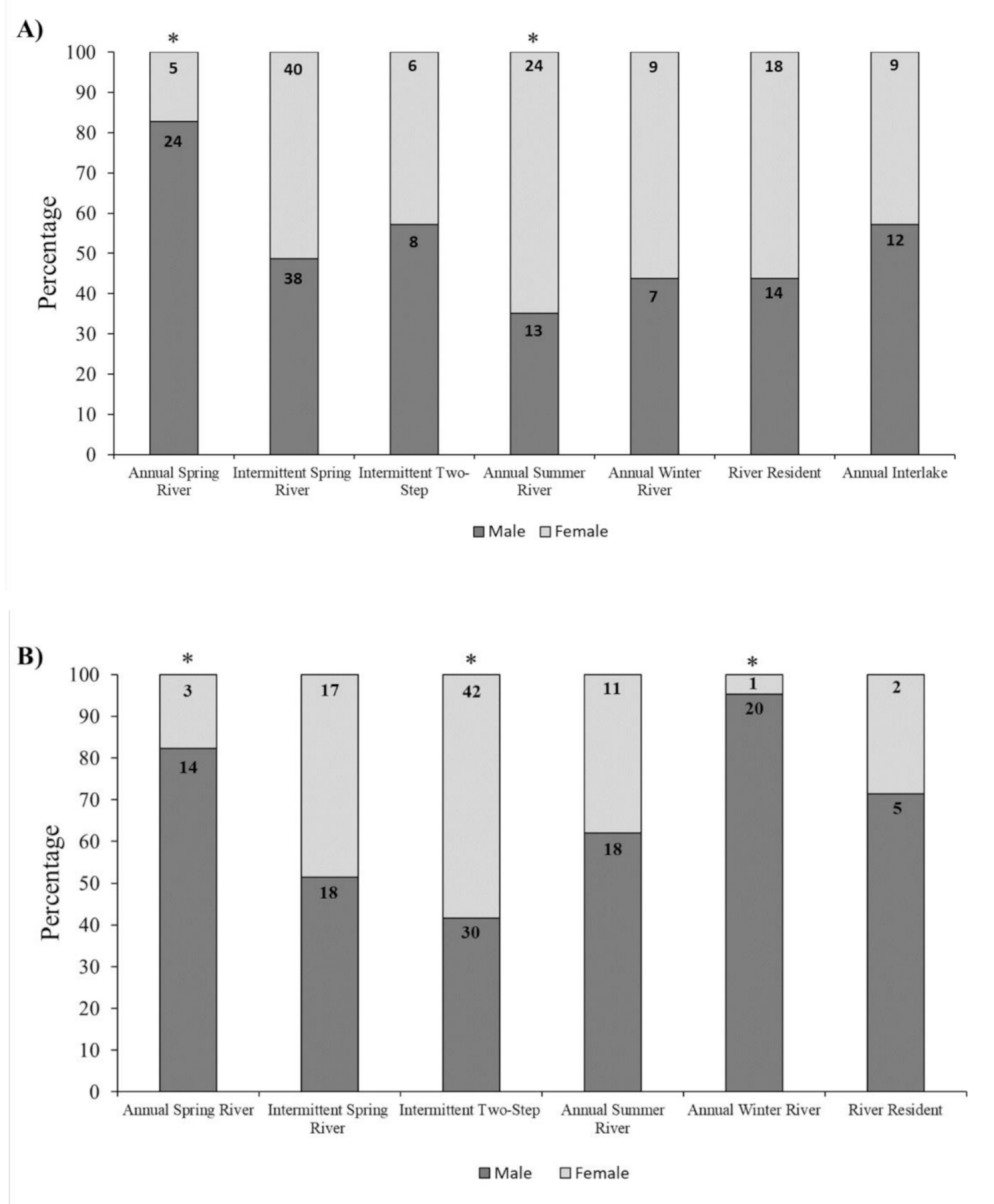
Sex ratios by migratory behavior for the **(A)** Huron-Erie Corridor and **(B)** Green Bay populations. Numbers inside each bar represent the total number of males or females for a given migratory behavior. Molecular sex was used when available for the Huron-Erie Corridor population (*N* = 201), and when unavailable, visual classifications were used by observing gametes during tag implantation (*N* = 26). All Green Bay individuals were classified visually (*N* = 181). Fish with an unknown sex via molecular or visual methods were not included in calculations (HEC, *N* = 11; GB, *N* = 4). Asterisks indicate differences in sex ratios when compared to the sex ratio of classified fish (Huron-Erie Corridor, 1.05:1, M: F, *N* = 227; Green Bay, 1.38:1, M: F, *N* = 181; Chi Square Goodness of Fit, α = 0.05)

All behaviors observed in the Huron-Erie Corridor and Green Bay populations included male and female individuals, but contrary to previous findings [11], sex ratios were unevenly distributed among migratory behaviors (Fig. 5). In both populations, a majority of annual spring river migrants were male (Huron-Erie Corridor, (χ² [1] = 11.7, *p* < 0.001); Green Bay, (χ² [1] = 4.14, *p* = 0.04). In the Huron-Erie Corridor, annual summer river migrants were disproportionally female (χ² [1] = 3.73, *p* = 0.05), but no differences were observed in any of the remaining migratory behaviors: intermittent spring river (χ² [1] = 0.16, *p* = 0.69), intermittent two-step (χ² [1] = 0.21, *p* = 0.65), annual winter river (χ² [1] = 0.34, *p* = 0.56), river residents (χ² [1] = 0.67, *p* = 0.41), or annual interlake (χ² [1] = 0.32, *p* = 0.57). In Green Bay, intermittent two-step migrants were disproportionally female (χ² [1] = 7.89, *p* < 0.01) and annual winter river migrants were disproportionally male (χ² [1] = 11.9, *p* < 0.001), but no differences in sex ratios were observed in remaining migratory behaviors: intermittent spring river (χ² [1] = 0.62, *p* < 0.43), annual summer river (χ² [1] = 0.20, *p* < 0.66), or river residents (χ² [1] = 0.52, *p* < 0.47).

## Discussion

Our cross-population analysis of long-term lake sturgeon movement patterns identified seven distinct migratory behaviors spanning spawning and other seasonal periods. All lake sturgeon migratory behaviors we observed have been previously identified [11, 39, 53, 54, 88, 89]. These behaviors included year-round river residency along with six patterns involving lake-river movements that varied in transition timing between habitats, migration periodicity, and the duration of time spent in each habitat. While the presence and frequency of migratory behaviors varied within and among populations, all six populations had multiple behaviors present (range, 2–7). Intermittent spring migrations were the only behavior common to all populations, whereas annual interlake migrations were unique to the Huron-Erie Corridor, which also showed the highest behavioral diversity. In contrast, the eastern Lake Superior population had the lowest behavioral diversity. Although the two most conventional behaviors, spring river (annual and intermittent) and intermittent two-step migrants [39, 46], were the most widespread; other strategies accounted for a substantial portion of individuals in some populations. Collectively, these results support the ideas that migratory diversity (1) is a general feature of lake sturgeon life history that is present in a wide range of environments, (2) may be shaped by the habitats available, their connectivity, and the resources they provide, and (3) may have important consequences for demographic and genetic connectivity within populations or metapopulations [82, 90]. Overall, lake sturgeon migratory diversity appears more extensive in the Great Lakes than previously recognized in studies limited to individual populations and/or shorter observations periods not encompassing full migration cycles.

Habitat diversity and productivity can influence the evolution of migratory strategies [1] and affect the spatial structuring of fish populations [91]. For example, the Huron-Erie Corridor and Green Bay supported lake sturgeon populations with the greatest migratory diversity (*N* = 7 and 6 behaviors, respectively) and most complex contingent structures. These regions contain estuary-like habitats, multiple spawning rivers, and extensive areas with productive nearshore lake habitats, with the Huron-Erie Corridor featuring Lake St. Clair, a unique lacustrine habitat juxtaposed between two large rivers. In contrast, the remaining four populations showed less migratory diversity (*N* = 2–4 migratory behaviors). These regions typically had one predominant spawning river, and three of four populations spawned in tributaries to Lake Superior where the shallow, nearshore habitat lake sturgeon prefer is limited [48, 92]. River residents and annual summer or winter river migrants primarily occurred in large river systems (Detroit, St. Clair, Menominee, Niagara, St. Marys, and St. Louis), which offer sufficient resources to support year-round or extended seasonal residency [93]. This link between habitat and behavior was most evident in the St. Louis River Estuary population, which was reintroduced with individuals sourced primarily from the Wolf River who were naïve to this new environment [94, 95]. The productive estuary-like habitats supported nearly half of categorized individuals as river residents, a much higher proportion than other populations, and likely reduced their need to enter the cold, low-productivity waters of Lake Superior. The presence of river residents in four of six populations and within highly fragmented river systems provides additional support that (1) partial migration in lake sturgeon is more widespread than previously thought and (2) individuals can permanently reside in relatively small, impounded river sections [96].

Habitat connectivity also directly affects migrating individuals [97, 98] and shapes the range of behaviors that can be expressed. The Huron-Erie Corridor, in contrast to other systems in this study, lacks barriers to adult movement, so sturgeon are more likely to express the phenotypic diversity characteristic of unperturbed populations. For example, up- and down-stream migrations to overwintering sites in Lake St. Clair preceded spawning for several individuals. Blocking these types of movements not only impedes access to important feeding or seasonal habitats but may also disrupt spawning. The complex, interconnected habitat of the Huron-Erie Corridor explains why we observed a unique migratory strategy (annual interlake migrants) and a distinct river/lake use pattern (overwintering in a lake during two-step migrations) not seen in any other population. Additional studies could verify if these behaviors also occur in populations that occupy other structurally complex ecosystems like the St. Marys and St. Lawrence rivers. Genetic data suggested that individuals from eastern Lake Superior and the north channel of Lake Huron comprised a metapopulation [71], but the connection between these regions is now partially blocked which limits movement between the upper and lower St. Marys River [88]. The presence of fish residing in the upper river during the summer suggests that migratory strategies including extended periods of river residence or interlake movements may have been more common in the St. Marys prior to fragmentation.

**Fig. 6:**
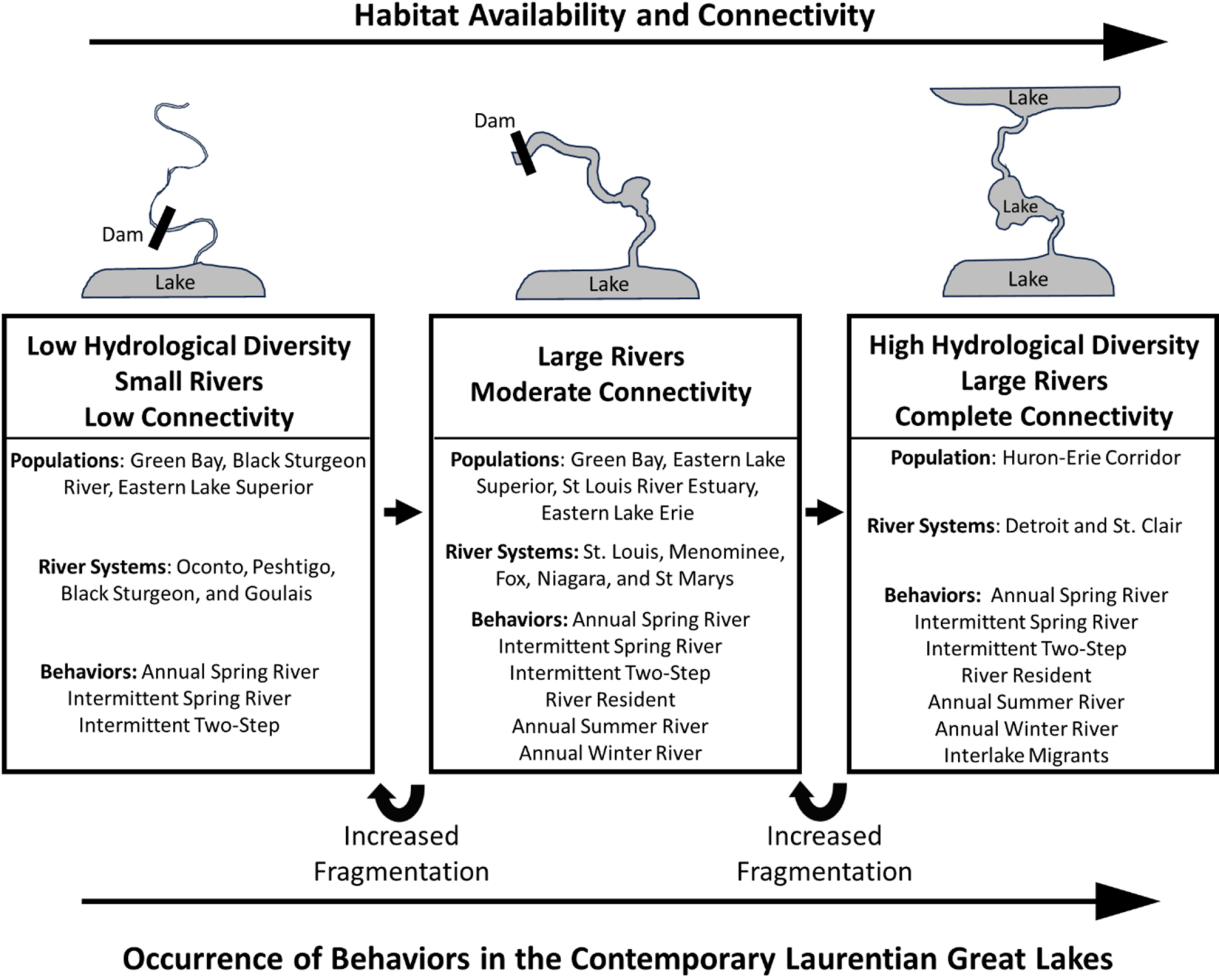
Proposed conceptual model of lake sturgeon migratory behavior in the Laurentian Great Lakes illustrating how habitat availability and connectivity are positively associated with migratory diversity. Systems are separated into three categories based on hydrological diversity and connectivity: (1) Low hydrological diversity, small rivers, low connectivity; (2) large rivers, moderate connectivity; and (3) high hydrological diversity, large rivers, no barriers to fish movement. Each study system and their associated rivers are assigned into these categories to highlight the relationships between habitat characteristics and presence of distinct migratory strategies observed in this study. Study populations may span multiple categories due to variation among river systems encompassed within their respective geographic region (e.g., Green Bay and eastern Lake Superior populations). This framework links habitat features to migratory behaviors and predicts behavioral shifts caused by increased habitat fragmentation

Our study is the first to concurrently describe and compare the intra- and inter- population variability in previously identified lake sturgeon migration patterns. Based on our results, we propose a conceptual model of lake sturgeon migratory behavior that hypothesizes lake sturgeon migratory diversity in the Great Lakes is associated with habitat availability, connectivity, and system productivity (Fig. 6). Key elements of habitat availability include the size (discharge) and connectivity of the spawning river, as well as the degree of hydrological diversity within the spawning river and adjoining lakes, which is influenced by the geographical organization of these habitats. Estuaries, deltas, and river reaches with lake-like characteristics (e.g., St. Louis River Estuary) are examples of features that add environmental complexity and productivity to the river-lake ecosystems lake sturgeon inhabit. Annual and intermittent spring river migrants along with intermittent two-step migrants will be common in most, if not all environments, whereas river residents, annual summer, and annual winter river migrants will occur in systems with greater hydrological diversity, connectivity, and in large rivers (Fig. 6). Interlake migrants may only occur in systems with high hydrological diversity and complete connectivity (Fig. 6). Increased fragmentation is expected to reduce migratory diversity by restricting expression of certain behaviors. Future empirical studies could be conducted to test the preceding predictions, which would help validate, refine, or revise our proposed conceptual model (Fig. 6).

Acoustic telemetry expanded our understanding of specific attributes shaping lake sturgeon migratory behaviors in the Great Lakes. Two-step migrations occur in multiple sturgeon species [99, 100], including lake sturgeon [88, 101–103], and by detailing the migration periodicity, duration, number of habitat transitions, and locations of occurrence, our findings provide the most comprehensive description of this behavior in lake sturgeon to date. We identified novel patterns that diverge from the definition by Bemis and Kynard [39], including overwintering in a different lake rather than the spawning river and downstream instead of upstream migrations. Some individuals alternated roughly one-year periods between rivers and lakes, a behavior not previously documented. Although classified as intermittent two-step migrants based on similarities in the overall behavioral pattern, the limitation of telemetry data to confirm spawning during each river stay suggests this 50:50 lake: river pattern may represent a distinct migratory strategy. Multi-river migrations [54, 62] with consistent seasonal timing but different residence times in each river highlight the importance of different rivers for meeting resource needs beyond spawning.

Differential migration is a widespread phenomenon across a variety of taxa [8, 104, 105]. In our study, migratory behavior was not sex-dependent as both males and females showed all identified migratory behaviors, but certain behaviors had skewed sex ratios. In the Huron-Erie Corridor, sex ratio differences compared to Kessel et al. [11] likely reflect our delineation between annual and spring river migrants, along with advancements in molecular sexing. Annual spring river migrants in both the Huron-Erie Corridor and Green Bay were predominantly male, consistent with findings that males may spawn annually unlike females due to energetic costs associated with gamete production [46, 106]. Given the multi-year intervals between spawning events in female lake sturgeon [46] and the potential for annual spring migrations by intermittently spawning males, annual spring river migrations may be related to feeding [55, 82] or be driven by as-yet-unidentified social interactions [11, 52]. In Green Bay, 95% of annual winter river migrants were male. Because this behavior mirrors that of two-step migrants but differs in migration periodicity, it’s possible these males are undergoing annual two-step spawning migrations but spawning itself cannot be confirmed via telemetry data. Except for annual spring river migrants, sex ratios among migratory behaviors were inconsistent across the Huron-Erie Corridor and Green Bay populations. The reasons why annual summer river migrants in the Huron-Erie Corridor and intermittent two-step migrants in Green Bay skewed female remain unclear but may include differences between the sexes in the energetic cost of gonad development [39, 40]. Because sex data only allowed comparisons across migratory behaviors for two populations, our ability to use cross-population comparisons for inferences related to sex-dependent migratory behavior in different environments is limited. Our findings suggest that sex differences in migratory behavior may vary by population and across multiple migratory behaviors, but this observation could also be an artifact of sampling bias that influences which individuals are available for and vulnerable to capture and ultimately tagged [107, 108].

The results of this study also emphasize the value of tracking individual movements over ecologically relevant timescales [109, 110] to gain a more comprehensive understanding of a species’ behavioral ecology. By observing movements in the Huron-Erie Corridor for 8–10 years, we identified an additional migratory behavior and 13 additional contingents that were not classified using similar analyses with shorter time series [11]. The substantial increase in identified contingents in our study of the Huron-Erie Corridor population compared to Kessel et al., [11] can be attributed to several factors: (1) the division of Kessel et al.’s [11] “lake dominant migrants” into annual and intermittent spring migrants, (2) the identification of intermittent two-step migrants as an additional migratory behavior, and (3) expanded array coverage that allowed for greater spatial resolution in regional sequence data over longer timeframes.

Further progress in understanding lake sturgeon migration requires addressing what variables influence the observed patterns in migratory behavior and at what life stage these patterns appear. The genetic underpinnings responsible for migration behaviors remain largely unknown in many species [111, 112]. Differences in DNA methylation but not neutral markers were identified for spring river migrants that move up or downstream to spawn in the St. Clair River [113]. Comprehensive genetic comparisons across all migratory behaviors could reveal genomic regions linked to migration related traits [113], as observed in salmonids [114, 115]. The environmental cues that drive spring spawning [88, 116] and overwintering migrations [117, 118] are well documented, but the cues driving consistent, synchronous habitat transitions during summer and early fall for multiple migratory behaviors remain unknown. While adult movement patterns are well documented compared to other life stages [46, 50], it remains unclear at what life stage the observed migratory behaviors first appear. Subadult sturgeon have been observed migrating between rivers and lakes [52, 119], and in the Wolf River, some individuals remained in the river, entered a lake, or made “prepubescent spawning runs” resembling adult spawning migrations [119]. However, it is unclear whether these types of subadult movements translate into adult migration behaviors in ways comparable to our results.

Maintaining population diversity is a critical consideration for effective fish conservation and management [29, 120, 121]. Migratory diversity likely enhances population stability and resilience of lake sturgeon populations by distributing risk across population segments (i.e., the portfolio effect; [28, 122, 123]). The discovery of significant migratory diversity across populations suggests that successful rehabilitation of this species in the Great Lakes minimally requires conservation of the phenotypic diversity reported here. Given limited evidence for a genetic basis for this diversity [113], conserving or increasing habitat connectivity may be an important aspect to promote conservation and restoration success. Likewise, expanding connectivity among all habitats used for spawning and other purposes could be more important than increasing availability of a single habitat type (e.g., spawning habitat; [124]). The diversity within and among populations also suggests management strategies tailored for specific populations or contingents may be warranted, especially where individuals using different migratory strategies overlap in small, vulnerable areas. For instance, individuals from all six migratory behaviors in Green Bay use the Menominee River, while six of seven in the Huron-Erie Corridor rely on Lake St. Clair, especially as an overwintering habitat. The frequent spatiotemporal overlap of multiple behavioral groups within relatively confined systems suggests they are critically important habitats and may warrant specific management considerations for their protection. Effective conservation and management strategies could address habitat needs and promote diversity of lake sturgeon populations to enhance the resilience and adaptive capacity of this species.

Our cross-population comparison revealed novel insights into lake sturgeon migratory behavior but also highlighted limitations with our approach and raised new questions for future studies. For instance, focusing on broad geographic regions limited our ability to (1) assess seasonal fidelity to localized areas in rivers and lakes [52, 55] and (2) determine whether river residents show movement patterns within river environments similar to those of lake-river migrants [55, 103]. More extensive arrays or the use of positional telemetry may enable fine-scale movement analysis and identification of critical habitats that can be parsed based on individual characteristics like sex, migratory behavior, or genetic stock assignment. Additionally, if power to observe variation in migratory behaviors was directly related to sample size, it was perhaps not surprising to observe that the two best-sampled populations had the most behavioral diversity and that rare behaviors were not detected in the populations with the smallest sample sizes. A more quantitative assessment of habitat availability and connectivity would also clarify the role of these variables in shaping migratory strategies and provide the means to directly test our proposed conceptual model. However, consistent patterns in the presence/absence of behaviors in certain environments indirectly support the idea that these variables may shape the expression and frequency of migratory strategies. A key challenge in replicating our approach is reliably detecting transitions between regions of interest, particularly with spatiotemporal variability in receiver coverage. Meeting this challenge was largely achieved through the extensive GLATOS network, except in Buffalo Harbor and the upper Niagara River, where winter coverage gaps may have missed instances of winter river residence. Addressing these limitations in future studies will be critical for further refining our understanding of lake sturgeon migratory behavior.

## Conclusions

In this study, we used long-term acoustic telemetry data to characterize the frequency and distribution of lake sturgeon migratory patterns among six distinct populations in the Great Lakes that occupy environments that vary in habitat availability and connectivity. Seven distinct migratory behaviors were identified based on differential patterns of lake and river use, and the presence and frequency of these behaviors varied substantially among populations. Our results highlight the importance of a cross-population, cross-lake approach that uses data at ecologically relevant spatiotemporal scales to gain a better understanding of a species’ migratory dynamics. This approach yielded novel insights into lake sturgeon migratory behavior in the Great Lakes, revealing how migratory variation influences the spatial structuring of populations and how migratory behavior in lake sturgeon may be linked to habitat availability and connectivity.

## Electronic supplementary material

Below is the link to the electronic supplementary material.

Supplementary Material 1

Supplementary Material 2

Supplementary Material 3

Supplementary Material 4

Supplementary Material 5

Supplementary Material 6

Supplementary Material 7

Supplementary Material 8

Supplementary Material 9

Supplementary Material 10

Supplementary Material 11

Supplementary Material 12

## Data Availability

The dataset supporting the conclusions for the Huron-Erie Corridor population is available in the Science Base Catalog [125]. Because this paper is meta-analysis, the contributing agencies that provided data for the other five lake sturgeon populations investigated do not have the same data release policies, and therefore, these data are not publicly available.

## Abbreviations

GLATOS: Great Lakes Acoustic Telemetry Observation System
ASW: Average Silhouette Widths
LOCF: Last Observation Carried Forward
