# Supplementary material for: Lake sturgeon behavioral diversity in the Laurentian great lakes: migratory patterns across populations and habitats
Source: Mov Ecol. 2025 Oct 23;13:75. doi: 10.1186/s40462-025-00585-y (PMC12548266; doi:10.1186/s40462-025-00585-y)
Supplement: Supplementary file 4 — Supplementary Material 4 [file 40462_2025_585_MOESM4_ESM.docx]

Supplementary Methods:

***Habitat and Regional Receiver Classifications and Creating Daily Location Histories***

*Habitat Classification Exceptions*

The most downstream receiver at the mouth of the Black Sturgeon River was classified as lake to detect habitat transitions more accurately due to receivers in the surrounding waters of Black Bay being seasonal and often not deployed during fall and winter. In the upper St. Clair River, two lines of receivers were deployed where Lake Huron outflows into the St. Clair River, and the downstream line was classified as river and the upstream line as lake to accurately detect habitat transitions between these two regions. Like the St. Clair River, a receiver where Lake Superior outflows into the St. Marys River was classified as lake to detect transitions more accurately in this region.

*Regional Classifications*

Our regional classifications included four Great Lakes (Lake Superior, Lake Michigan, Lake Huron, or Lake Erie), Lake St. Clair, and multiple river systems (*Huron-Erie Corridor* - Detroit River, St. Clair River; *Green Bay* - Fox River, Menominee River, Oconto River, Peshtigo River, Cedar River, Ford River, Duck Creek, Pensaukee River, Suamico River, Little Suamico River, Rapid River; *St. Louis River Estuary* - St. Louis River, Kingsbury Creek, Nemadji River, Keweenaw Waterway, Ontonagon River, Black Sturgeon River; *Black Sturgeon River* – Black Sturgeon River; *eastern Lake Superior* - St. Marys River, Goulais River, and Batchawana River; *eastern Lake Erie* – Niagara River).

***Statistical Analyses***

*Daily Location History Timeframes for Each Population*

In the Huron-Erie Corridor, the analysis timeframe ranged from 1/1/2015 – 9/19/2023 due to the last group of sturgeon being tagged in 2015 and receiver coverage expanding during this period with the installation of grid arrays in Lake Erie, Lake St. Clair, and Lake Huron. Despite tagging and monitoring of Green Bay lake sturgeon beginning in 2011, we restricted statistical analysis to data collected between 6/29/2021 and 10/05/2023 to align with the deployment of a grid array. Prior to grid deployment, limited coverage at key geographic bottlenecks impeded consistent detection of spatial transitions and often violated LOCF assumptions; therefore, we excluded this data from statistical analysis. The extensive receiver coverage throughout the St. Louis River and Estuary and in Lake Superior just outside both river entrances allowed an analysis time frame from 4/16/2016 – 10/30/2023. For the Black Sturgeon River population, location histories ranged from 2/26/2018 – 10/20/2022 which minimized deployment gaps of multiple river receivers to ~1-2 weeks and aligned with continuous deployment of the two most downstream receivers that detected regional transitions between the Black Sturgeon River and Lake Superior. To coincide with consistent array coverage in Batchawana and Goulais bays as well as the St. Marys and Goulais rivers, sequences ranging from 1/01/2016 – 10/31/2022 were used for the eastern Lake Superior population. Extensive array coverage in Lake Erie and throughout the Huron-Erie Corridor allowed us to use entire detection histories for individuals from the eastern Lake Erie population, which ranged from 6/19/2014 – 10/04/2022.
