# Supplementary material for: Lake sturgeon behavioral diversity in the Laurentian great lakes: migratory patterns across populations and habitats
Source: Mov Ecol. 2025 Oct 23;13:75. doi: 10.1186/s40462-025-00585-y (PMC12548266; doi:10.1186/s40462-025-00585-y)
Supplement: Supplementary file 5 — Supplementary Material 5 [file 40462_2025_585_MOESM5_ESM.docx]

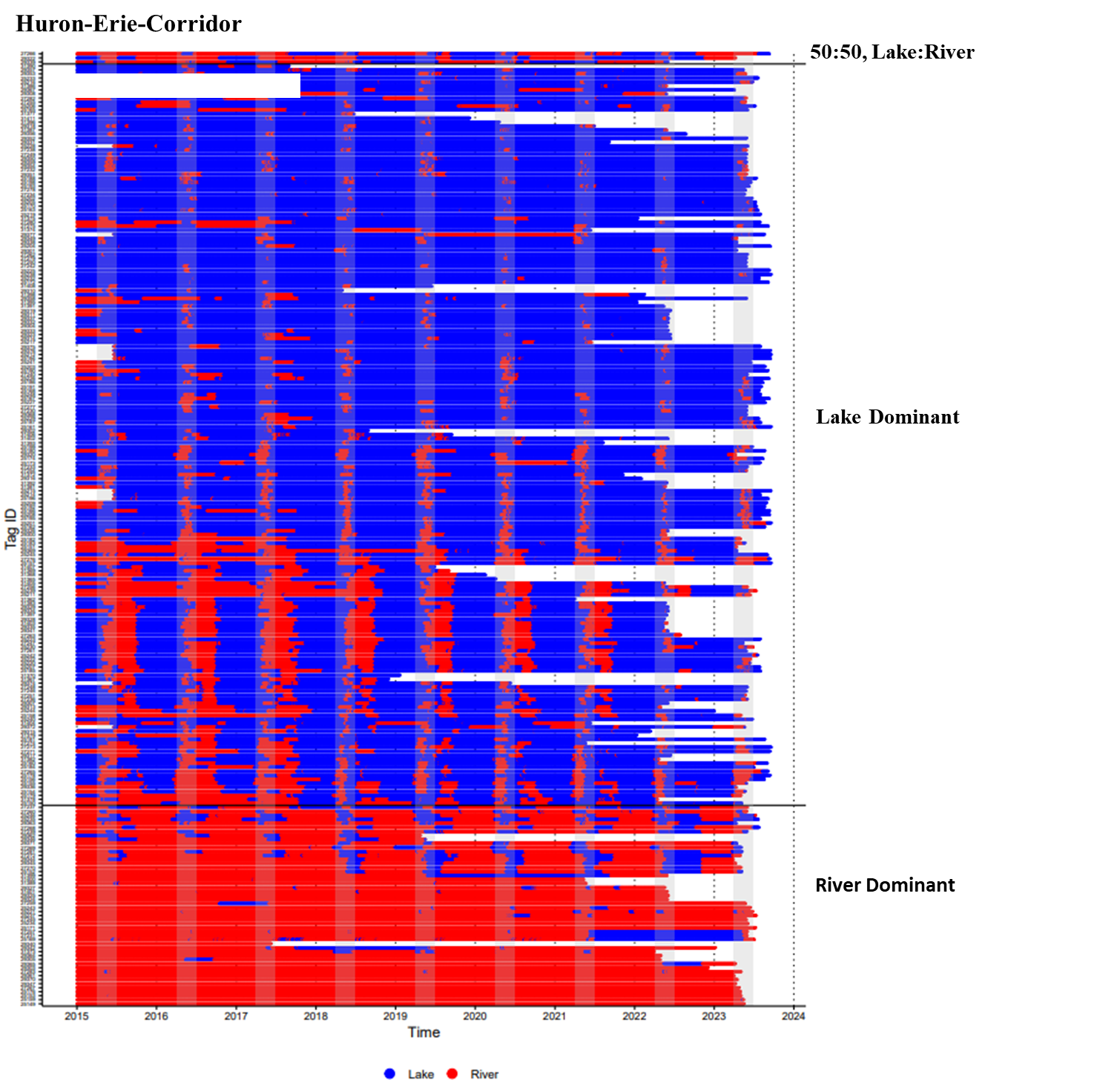


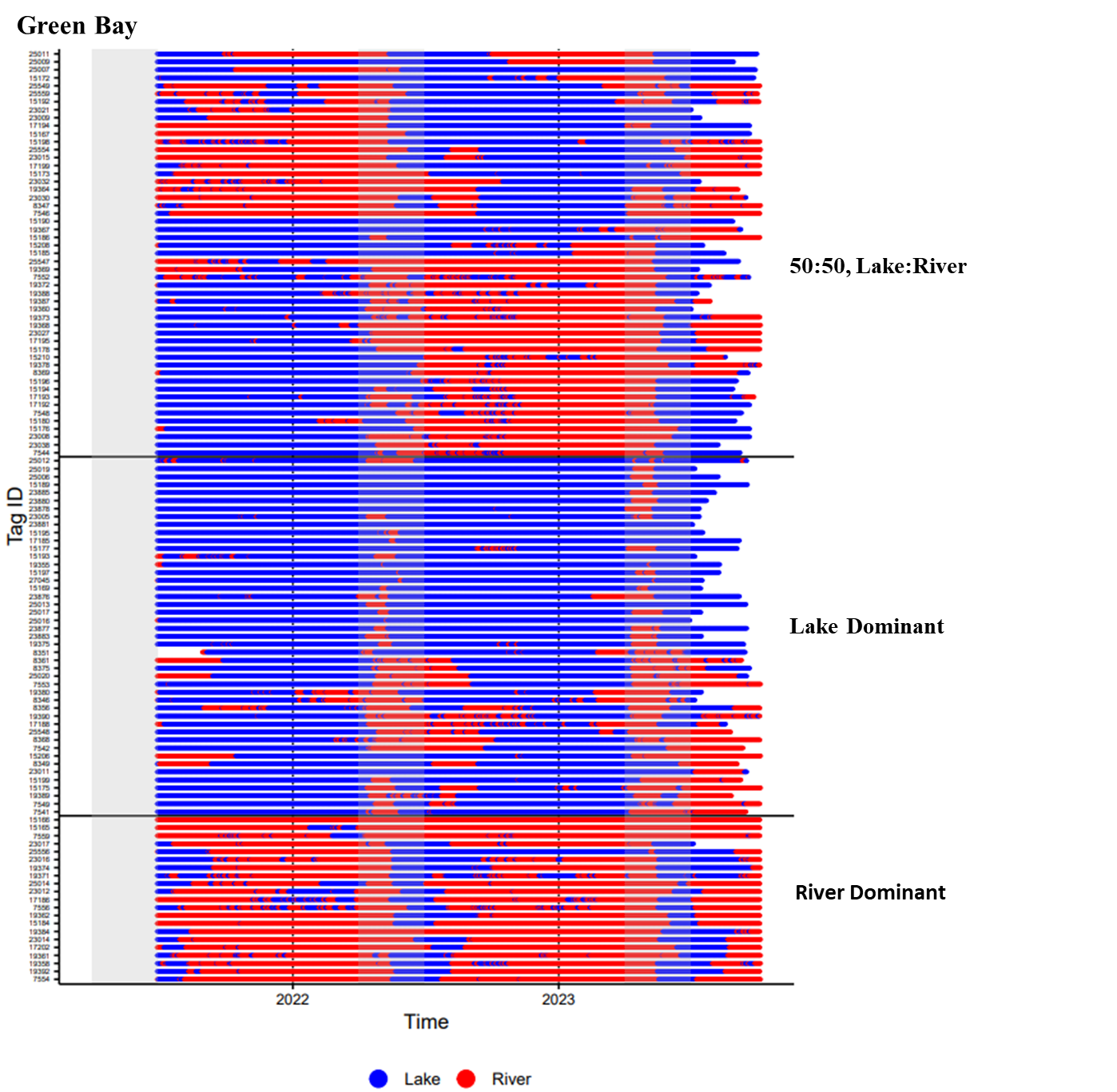


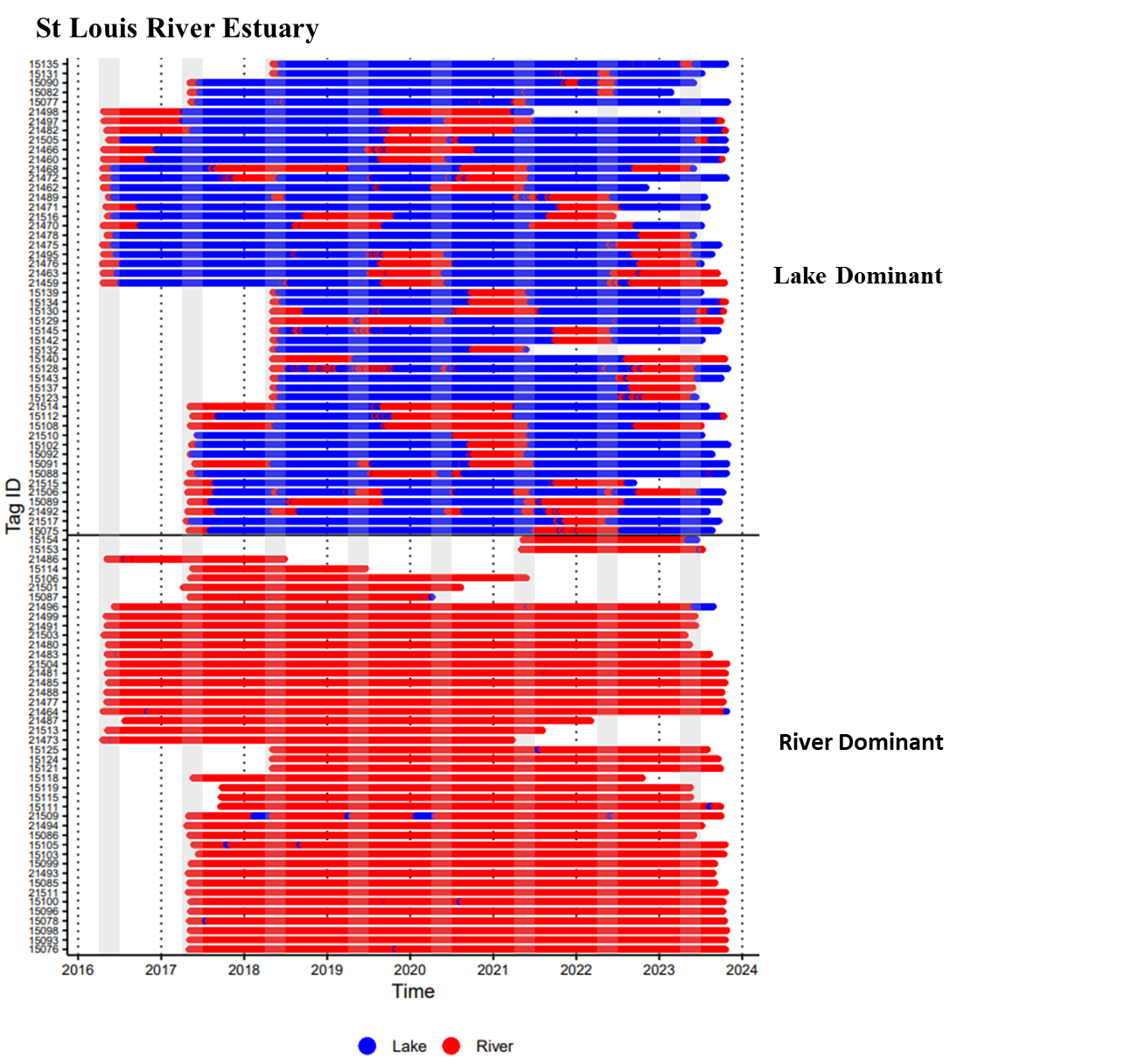


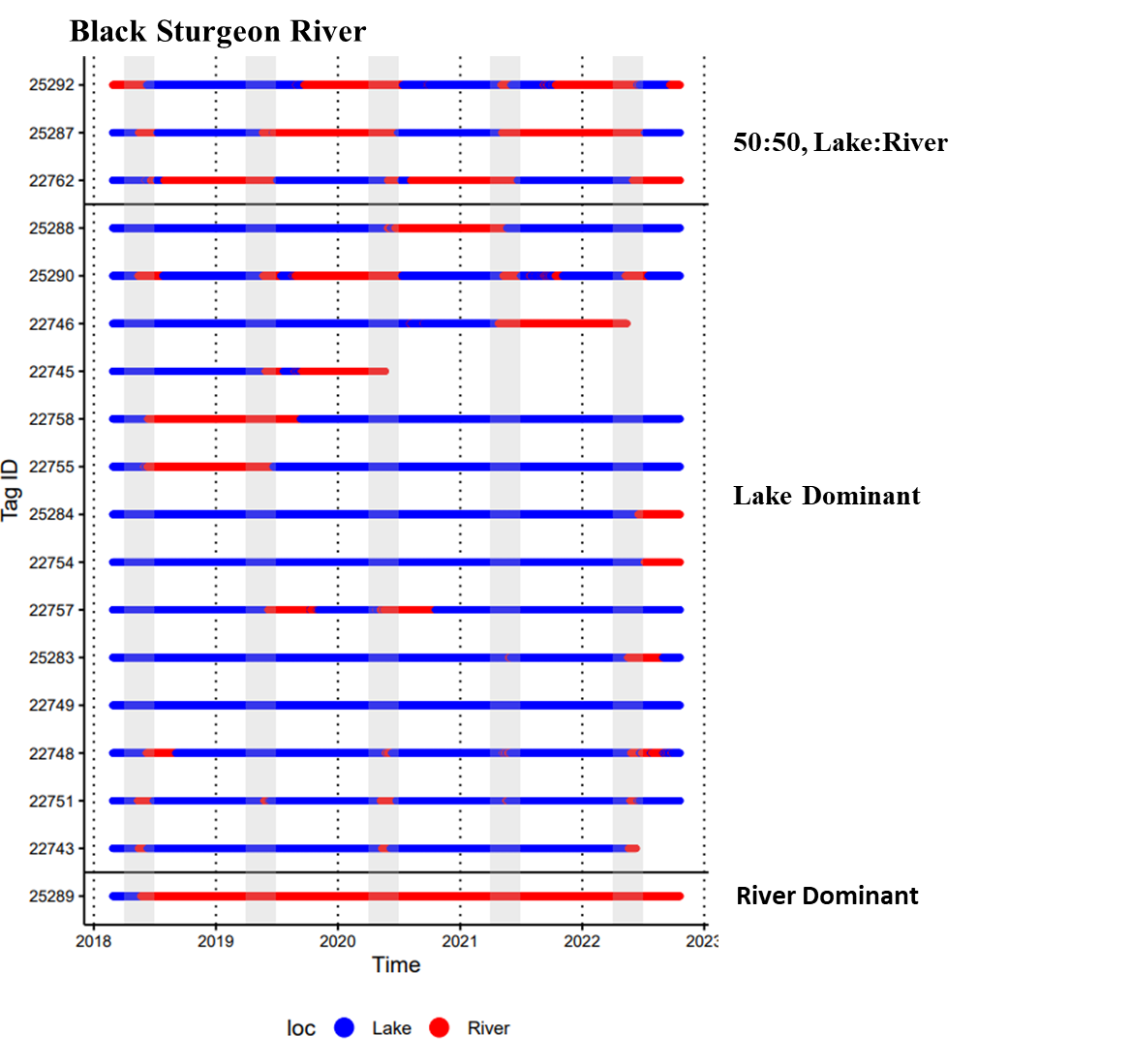


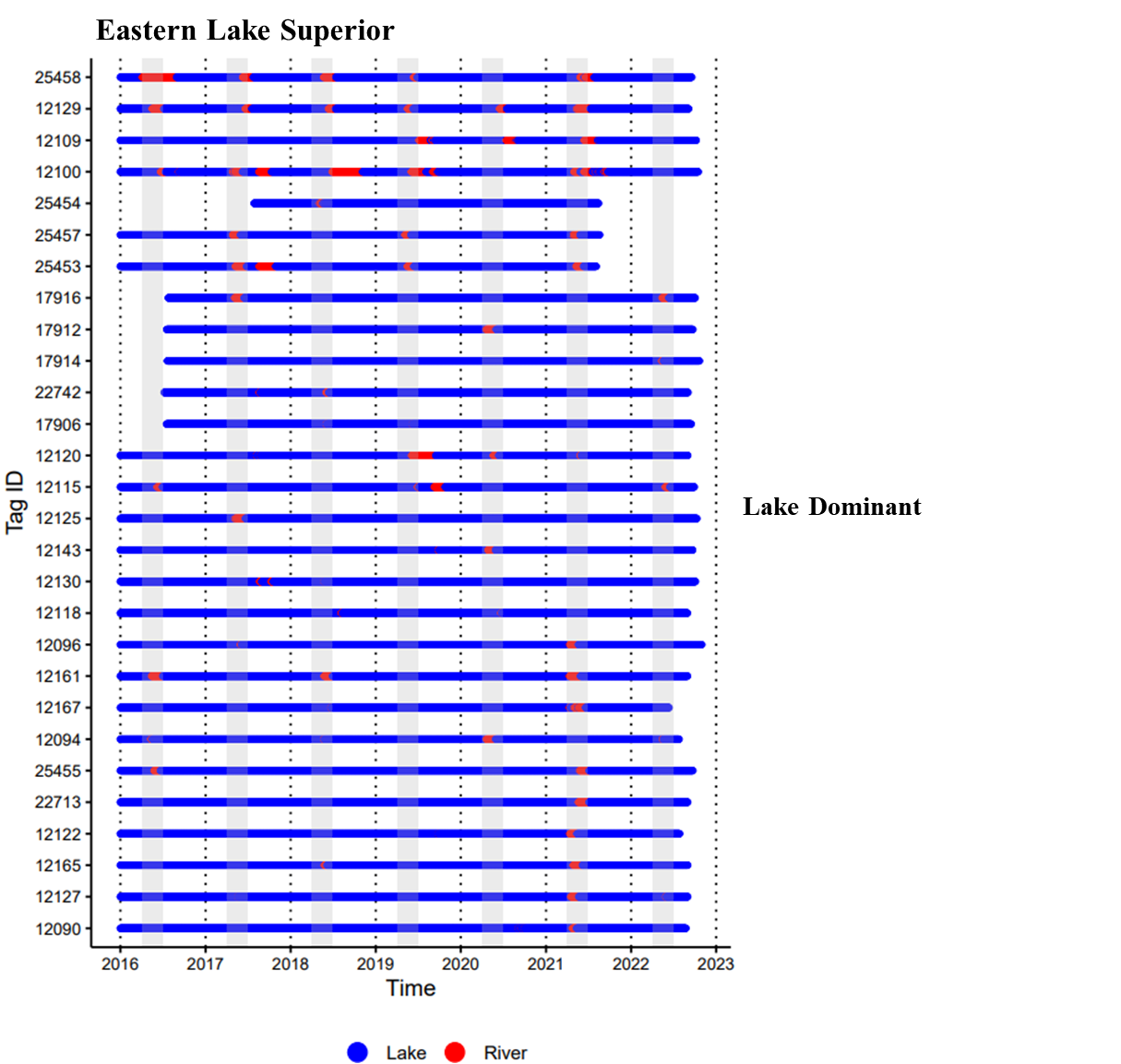


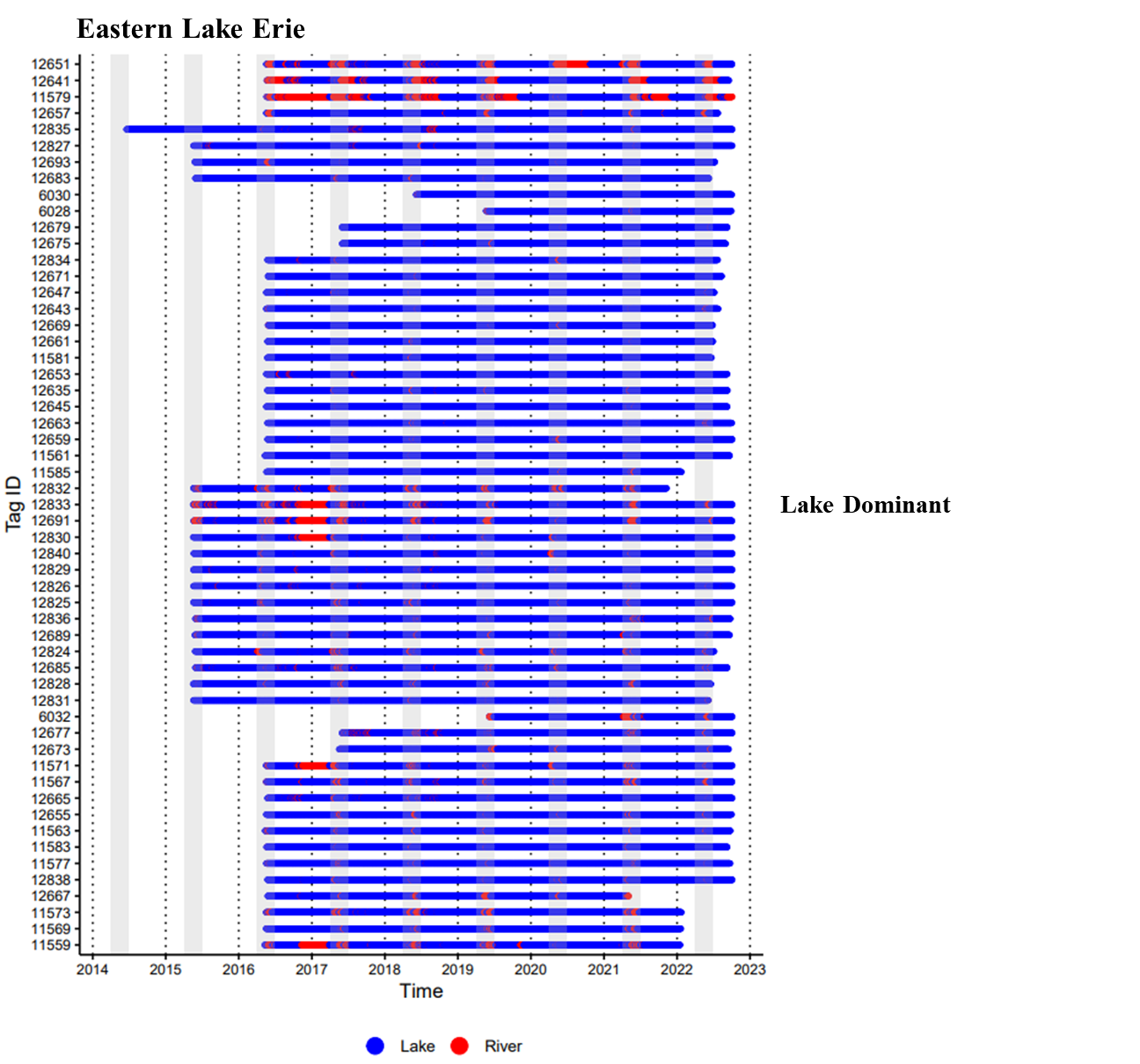


**Supplemental File 5.** Habitat-use patterns identified from agglomerative hierarchical clustering and visual inspections of habitat sequences for all six populations investigated. “Lake dominant” individuals primarily resided in lakes but were also detected in rivers, whereas “river dominant” individuals occupied rivers exclusively or were occasionally detected in lakes. “50:50 lake:river” individuals alternated approximately one-year periods between lake and river habitats. Solid horizontal lines delineate habitat use groups, and gray bars indicate the typical lake sturgeon spawning season in the Laurentian Great Lakes (April-June).
