# Supplementary material for: Lake sturgeon behavioral diversity in the Laurentian great lakes: migratory patterns across populations and habitats
Source: Mov Ecol. 2025 Oct 23;13:75. doi: 10.1186/s40462-025-00585-y (PMC12548266; doi:10.1186/s40462-025-00585-y)
Supplement: Supplementary file 6 — Supplementary Material 6 [file 40462_2025_585_MOESM6_ESM.docx]

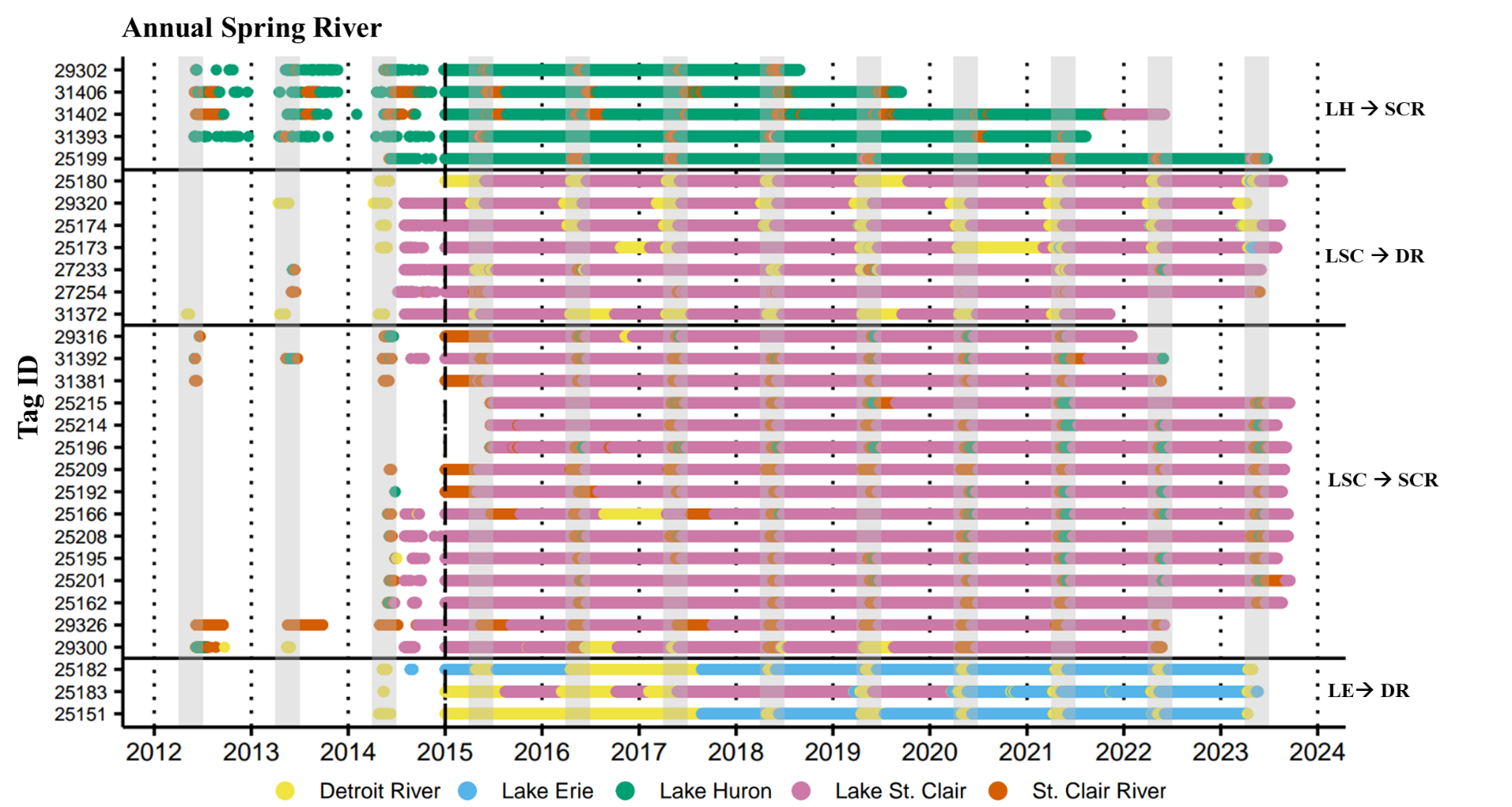


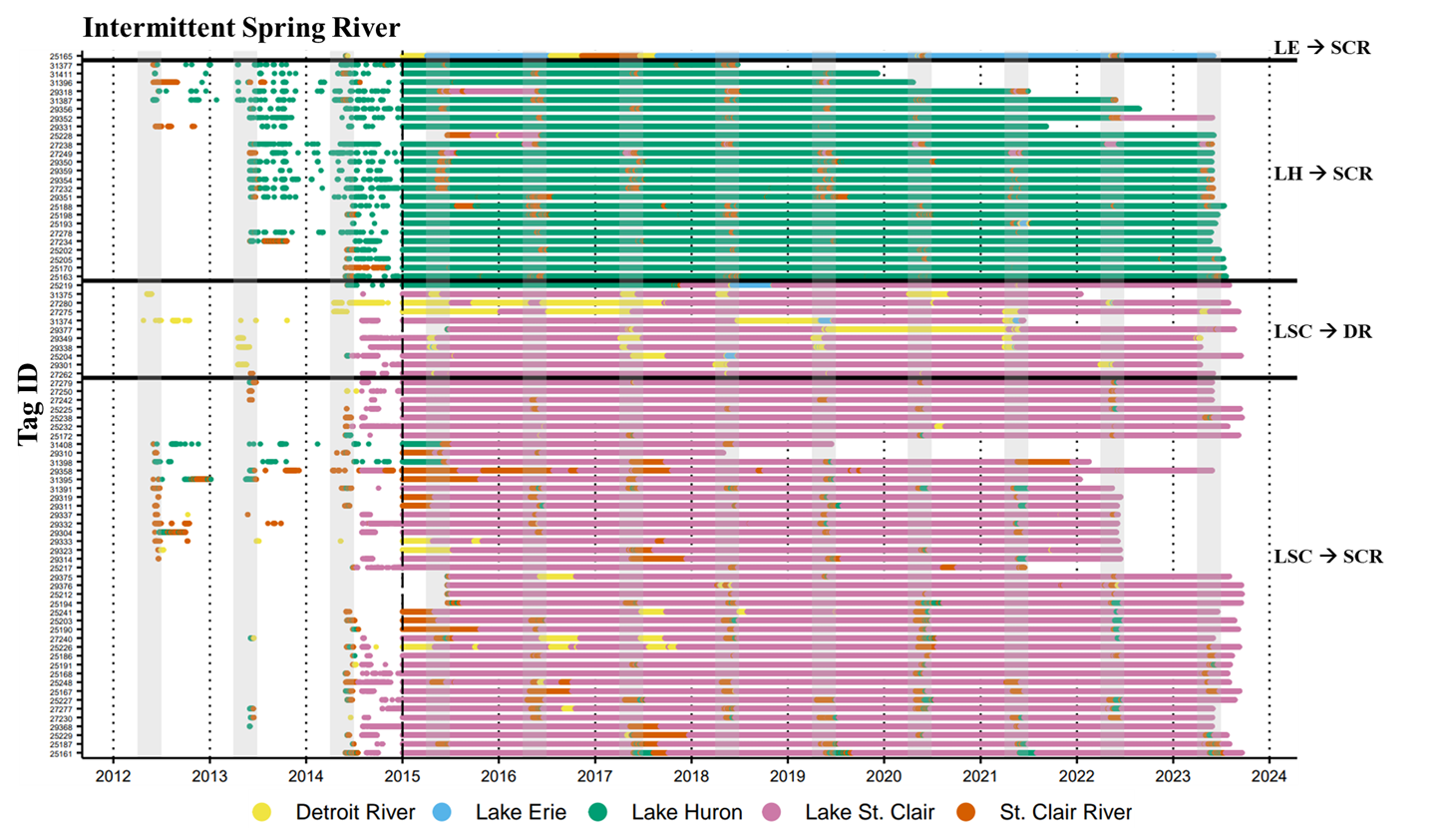


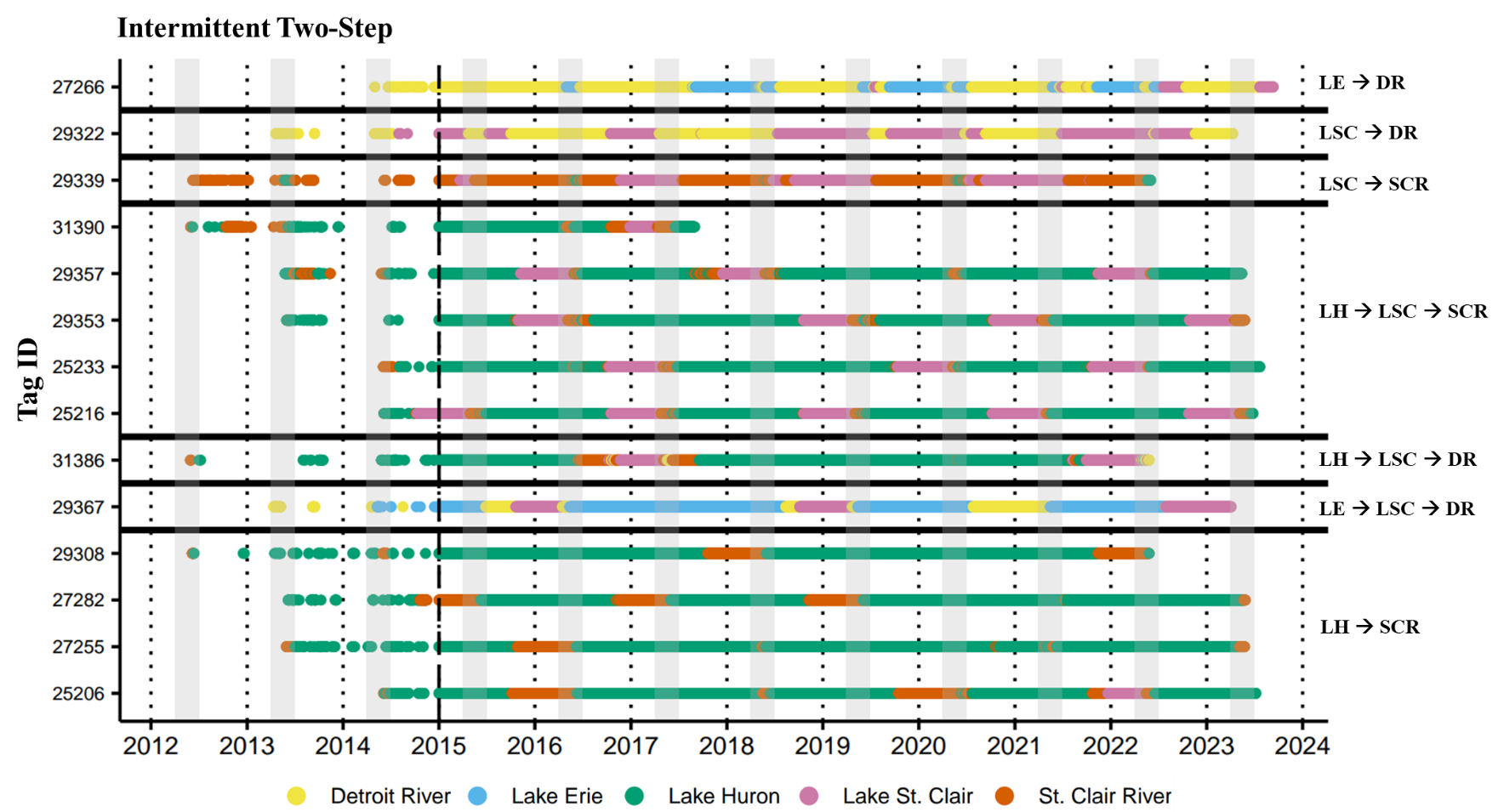


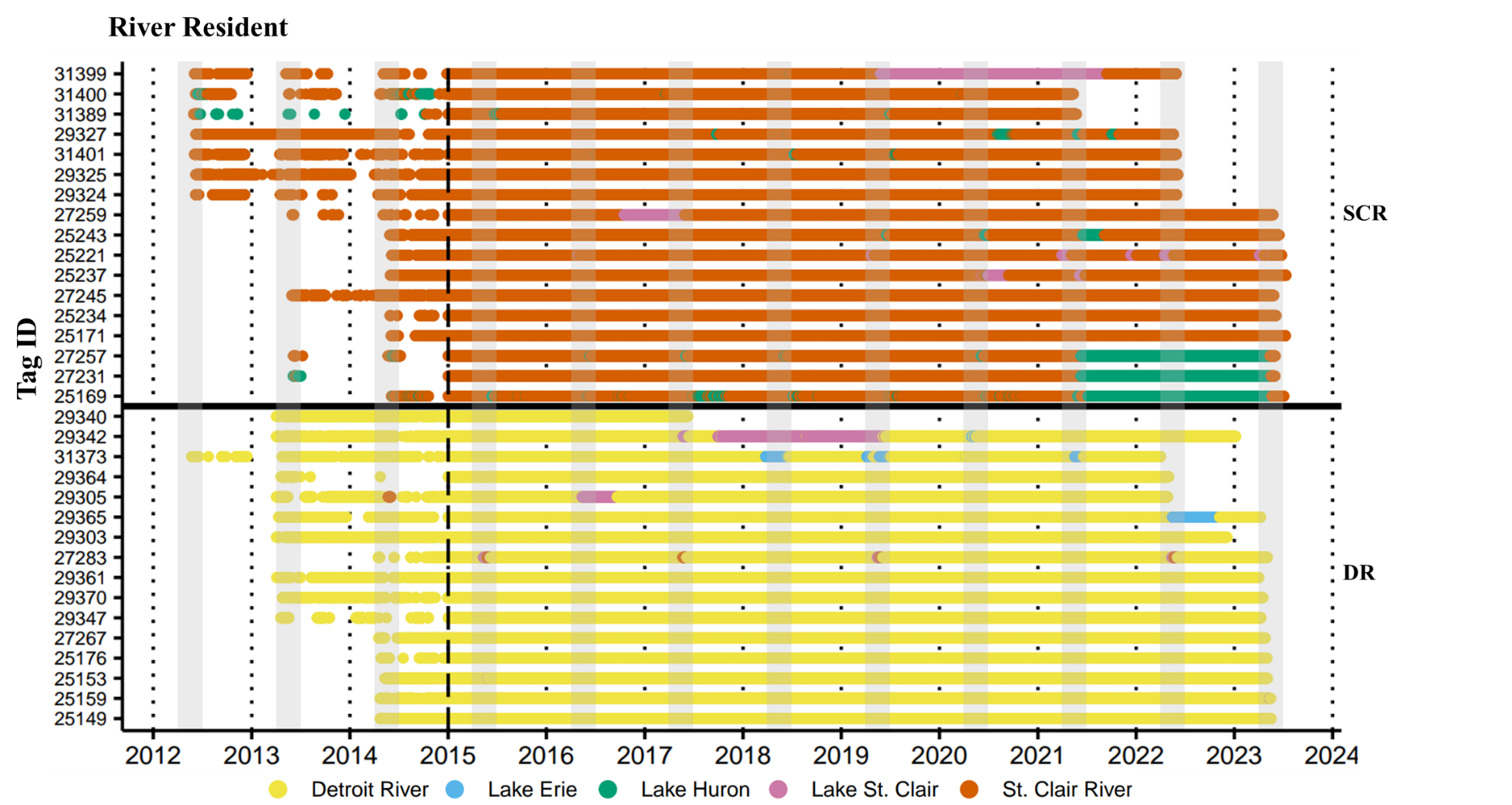


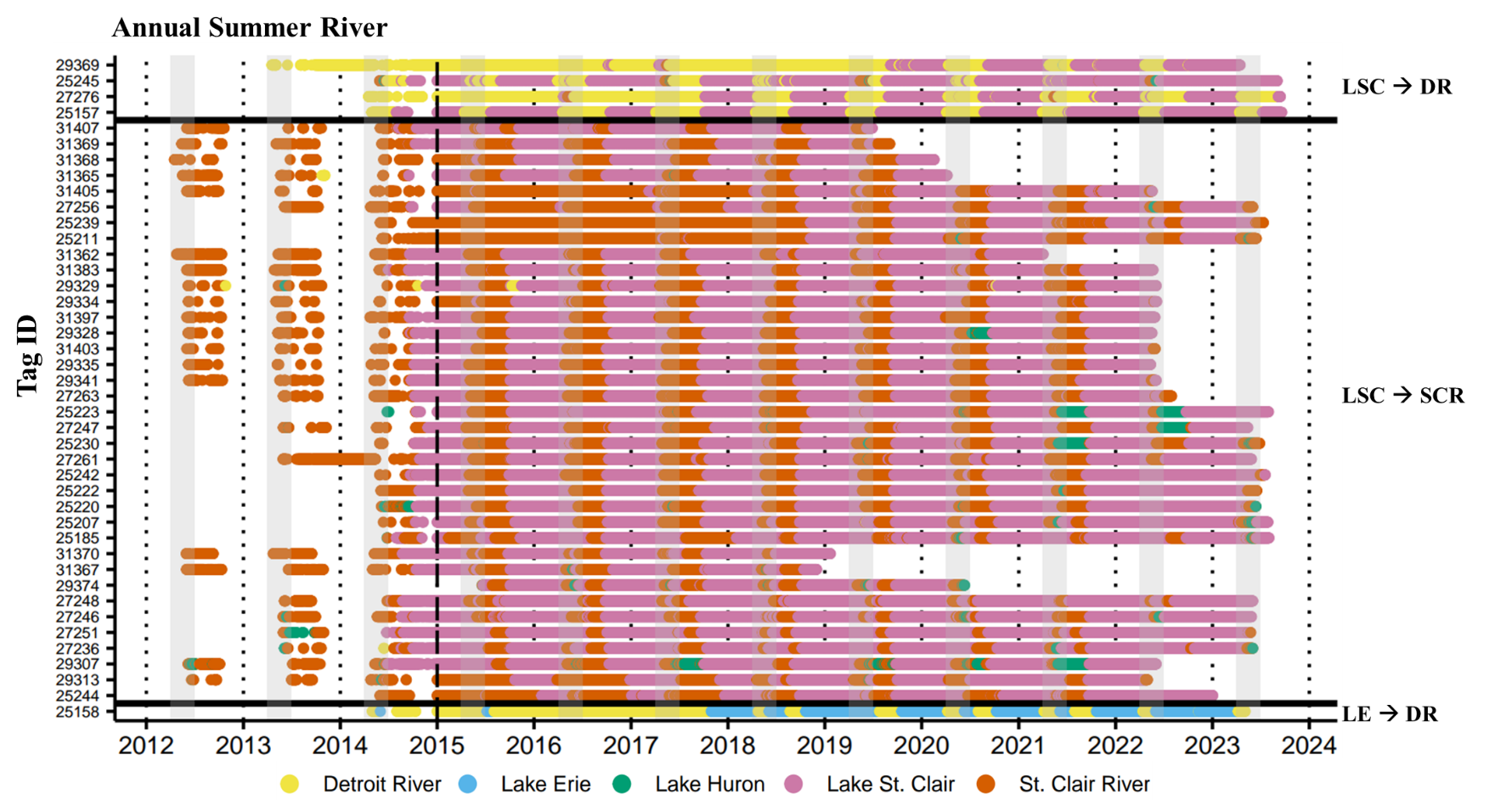


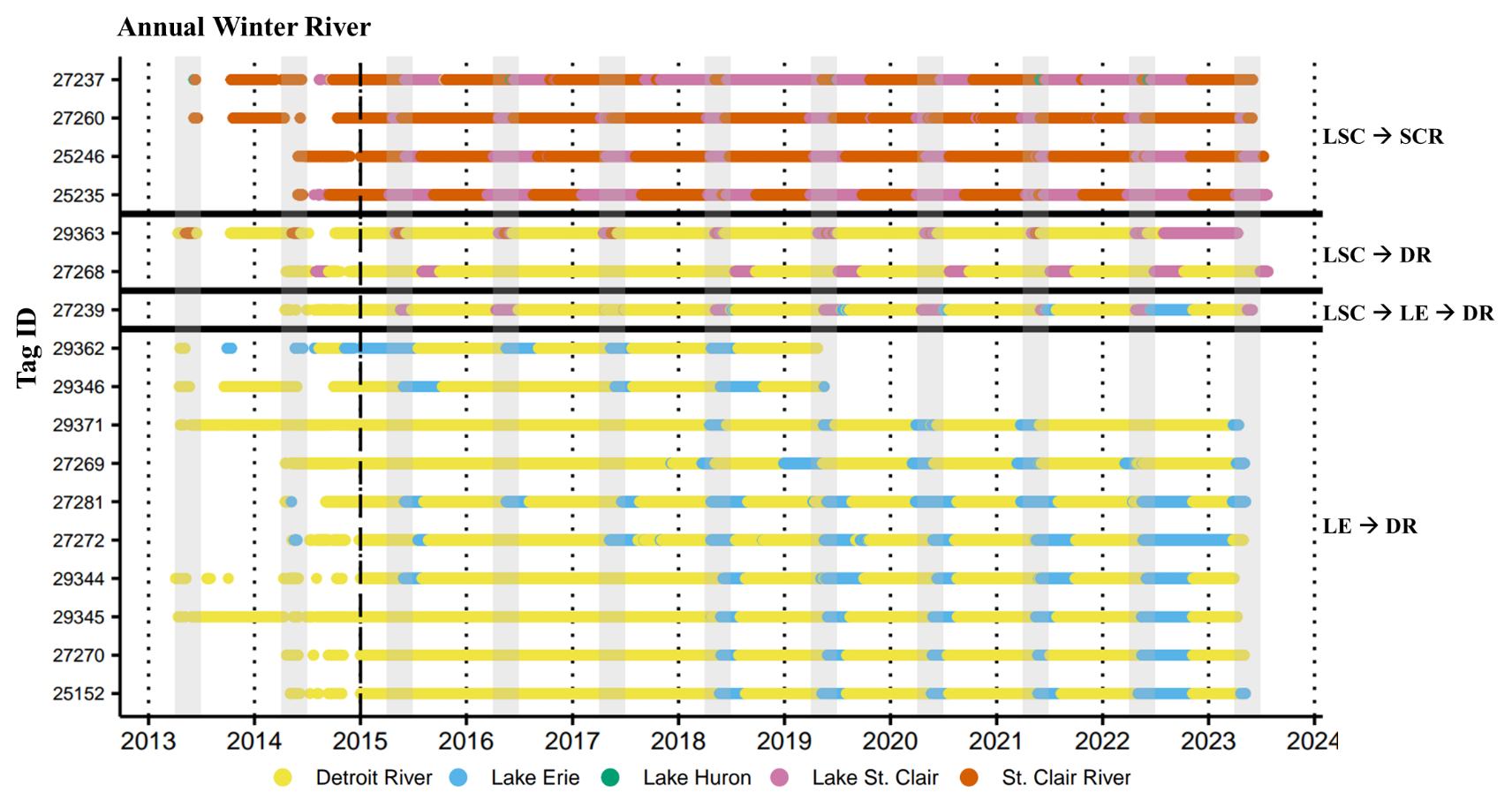


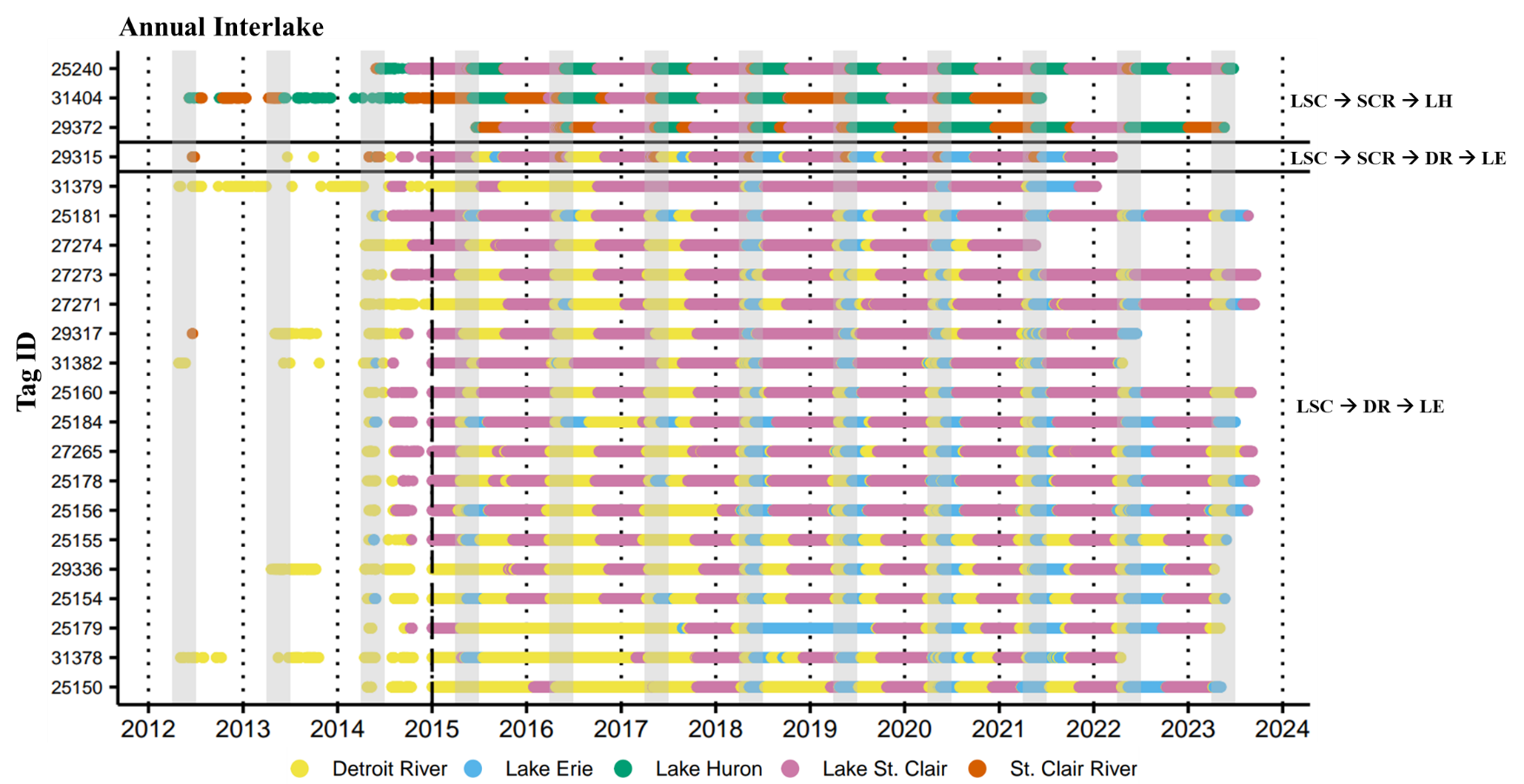


**Supplemental File 6.** Regional sequences displaying the assigned migratory behavior and contingent for all classified individuals (*N*=238) from the Huron-Erie Corridor population. Individuals were classified using agglomerative hierarchical clustering followed by visual inspection of habitat and regional sequences. Contingents are displayed on the right side of each migratory behavior plot, and all regional names used for contingents are abbreviated: Lk. St. Clair (LSC), Lk. Huron (LH)**,** Lk. Erie (LE), Detroit R. (DR), St. Clair R. (SCR). Solid horizontal lines delineate contingents, gray bars indicate the typical lake sturgeon spawning season in the Laurentian Great Lakes (April-June), and the vertical dashed line denotes the daily location history time frame used for sequence analyses (1/1/2015 – 9/19/2023). Gaps in sequences prior to the dashed line did not have last observation carried forward applied and therefore display data based solely on receiver detections.
