# Supplementary material for: Lake sturgeon behavioral diversity in the Laurentian great lakes: migratory patterns across populations and habitats
Source: Mov Ecol. 2025 Oct 23;13:75. doi: 10.1186/s40462-025-00585-y (PMC12548266; doi:10.1186/s40462-025-00585-y)
Supplement: Supplementary file 7 — Supplementary Material 7 [file 40462_2025_585_MOESM7_ESM.docx]

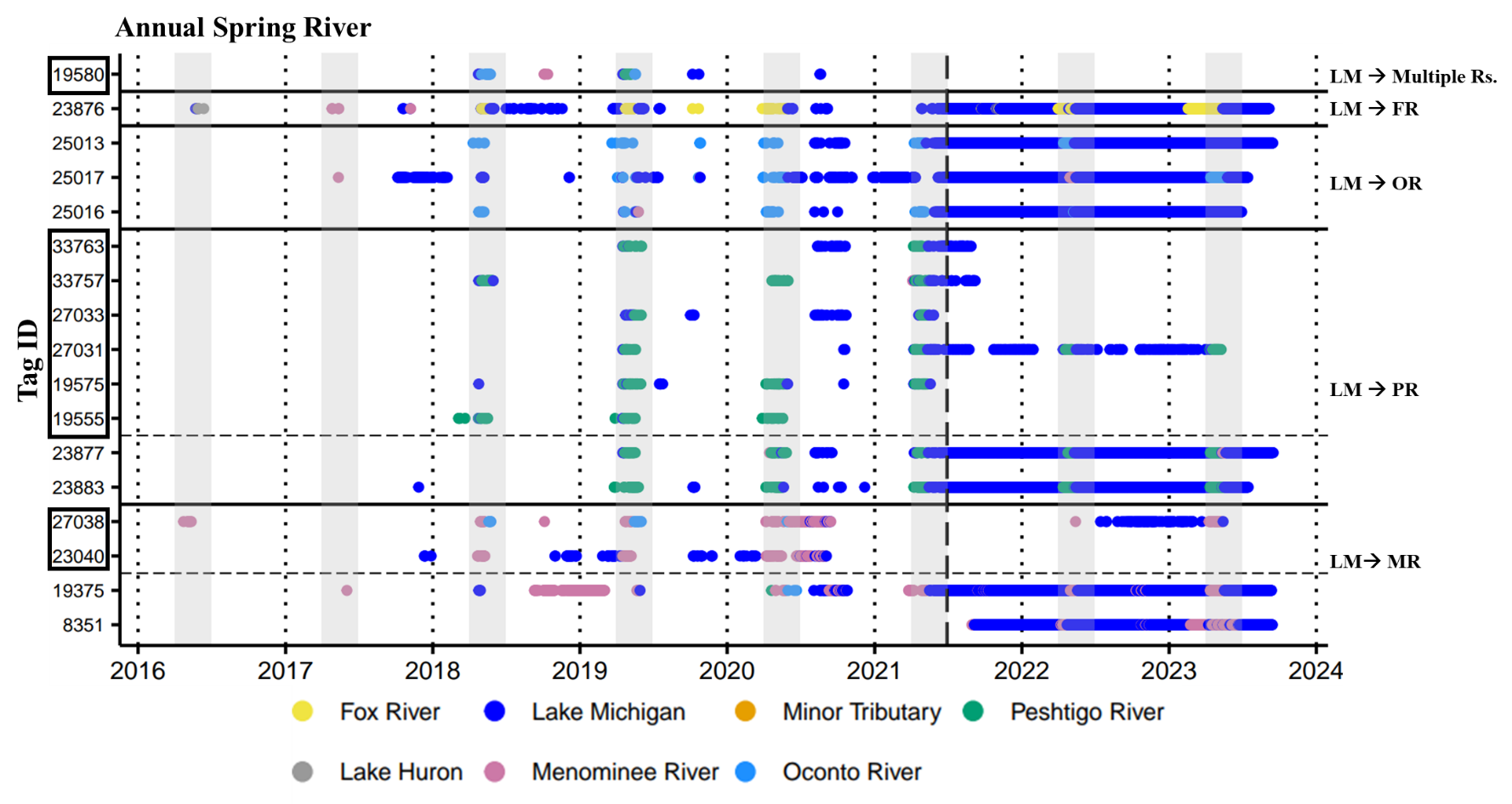


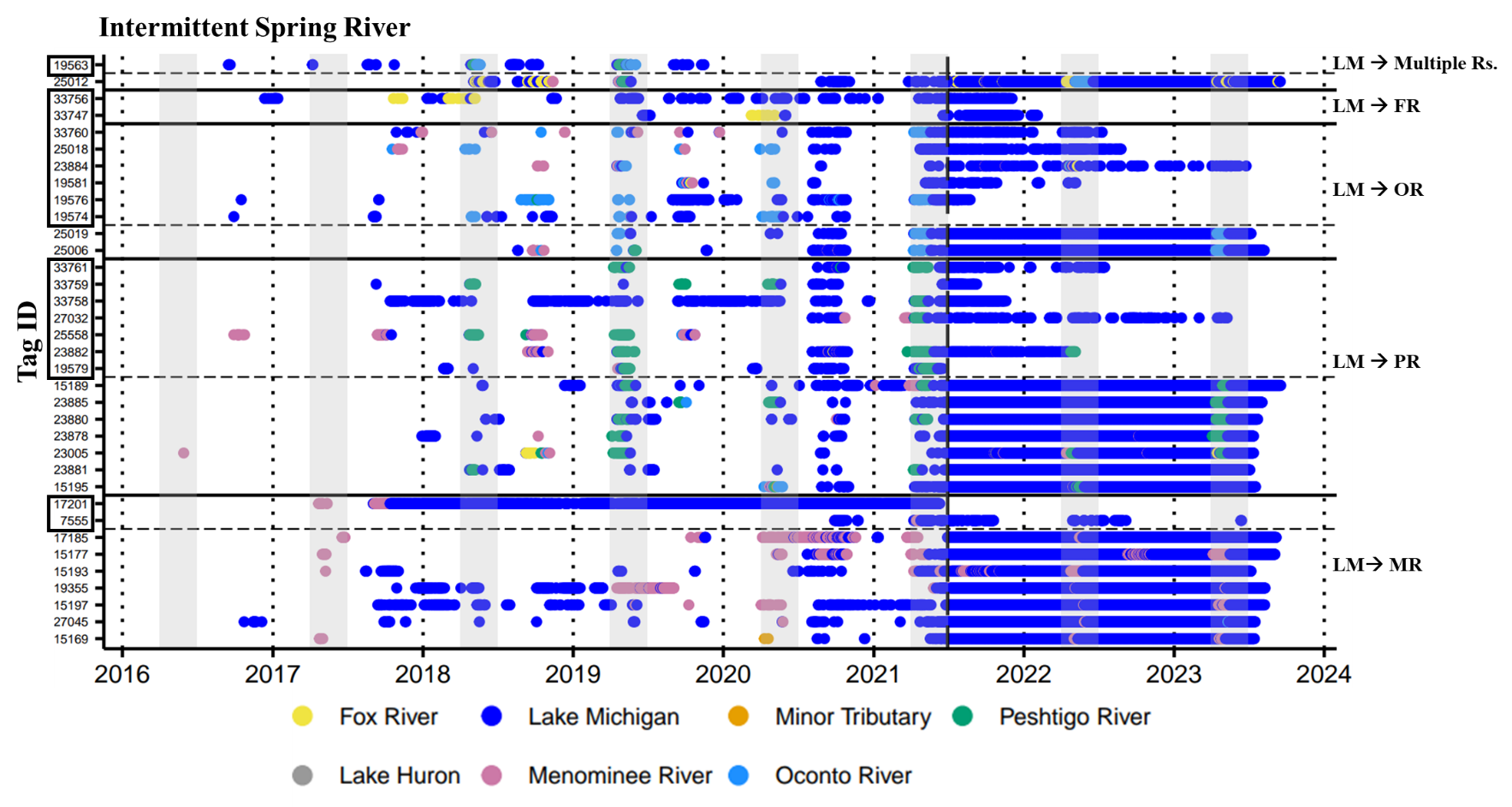


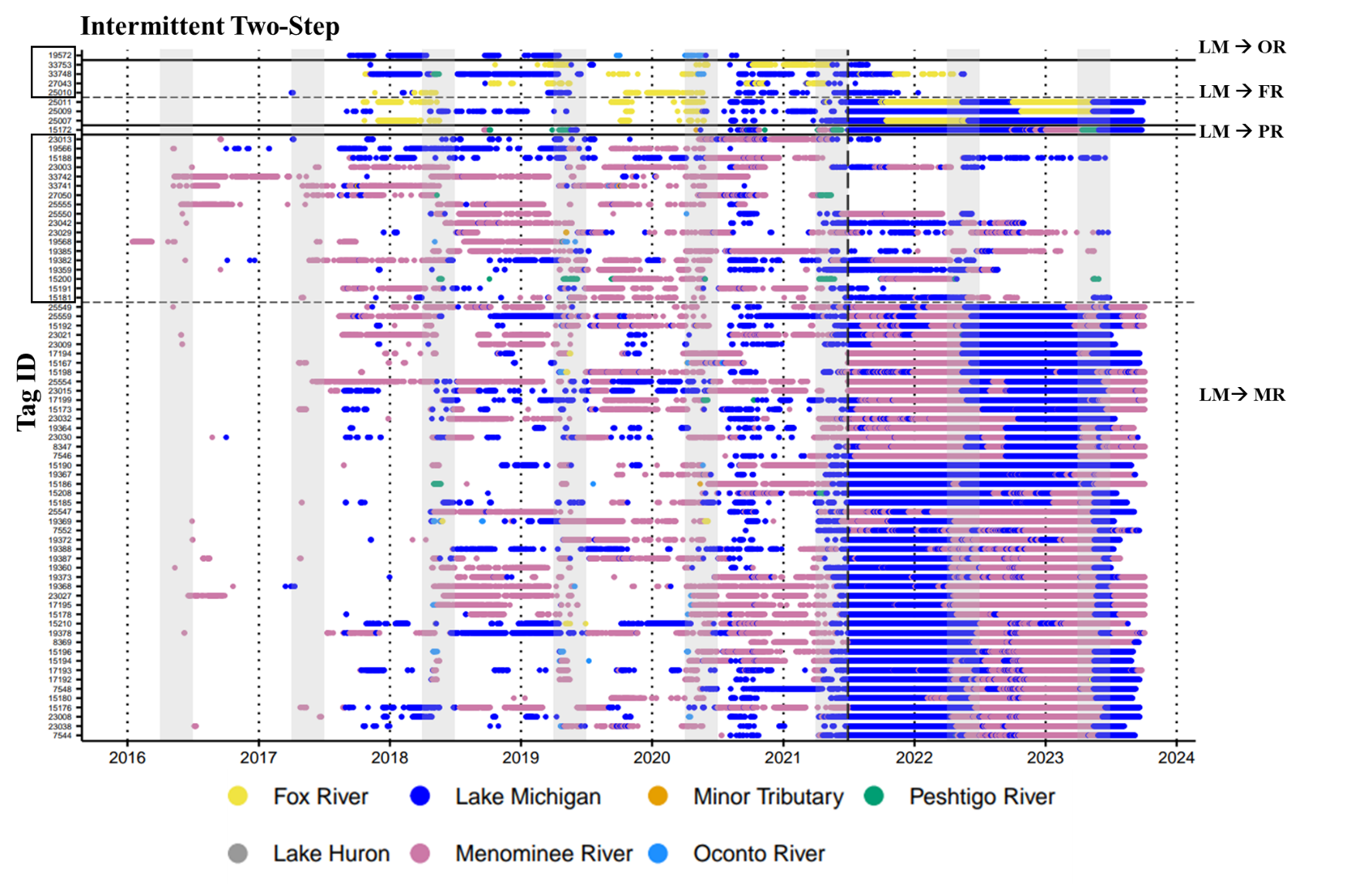


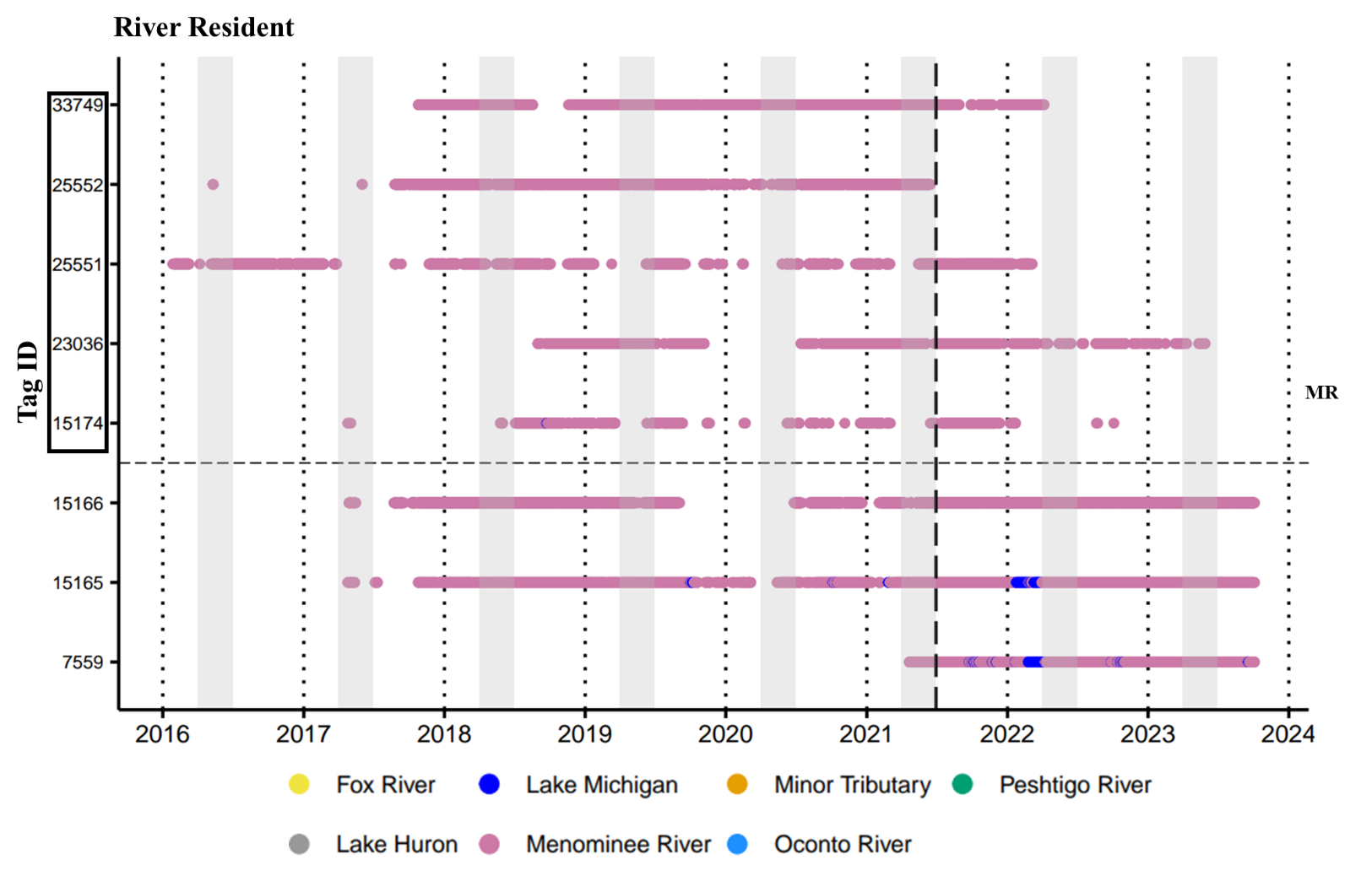


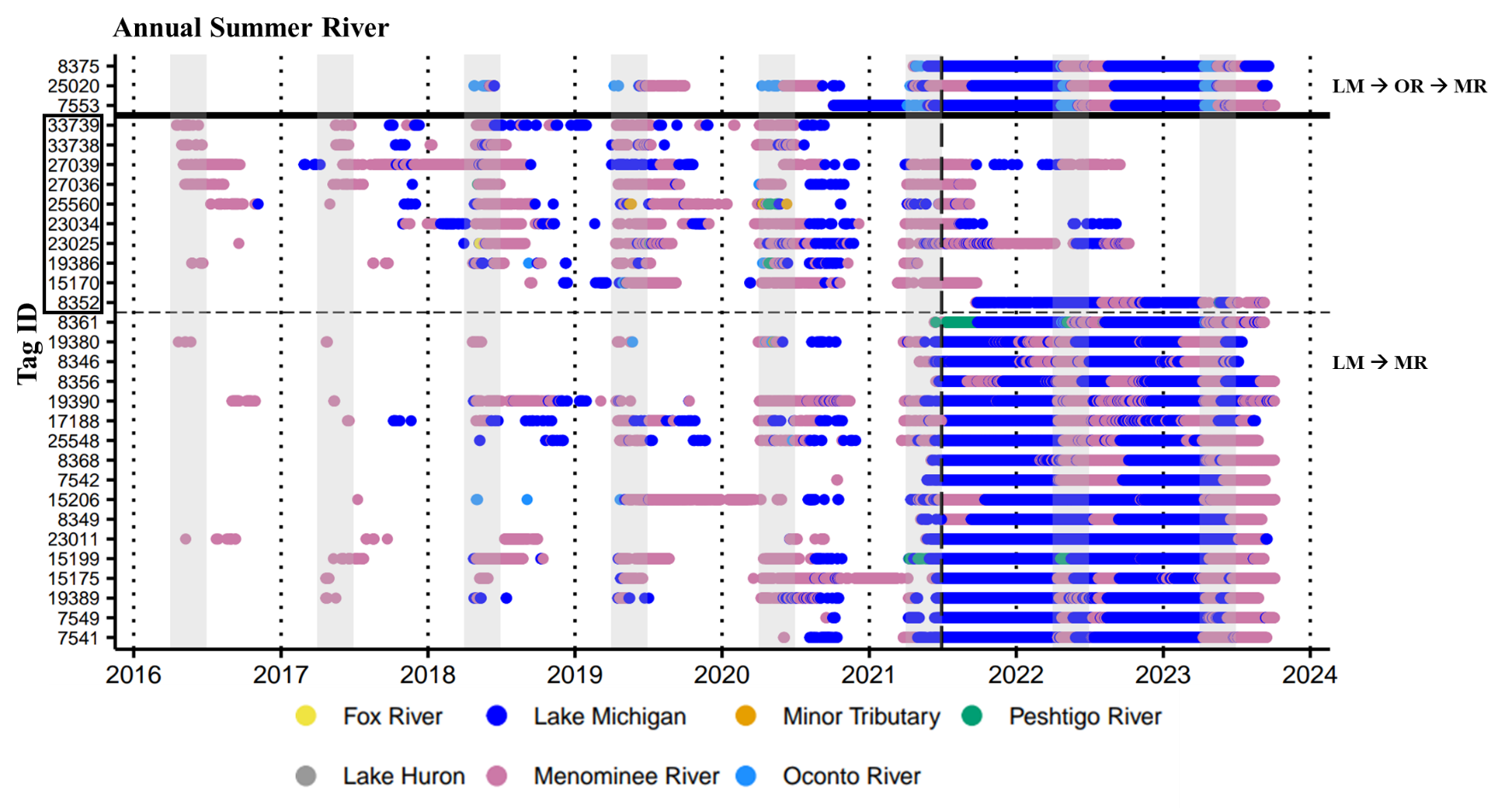


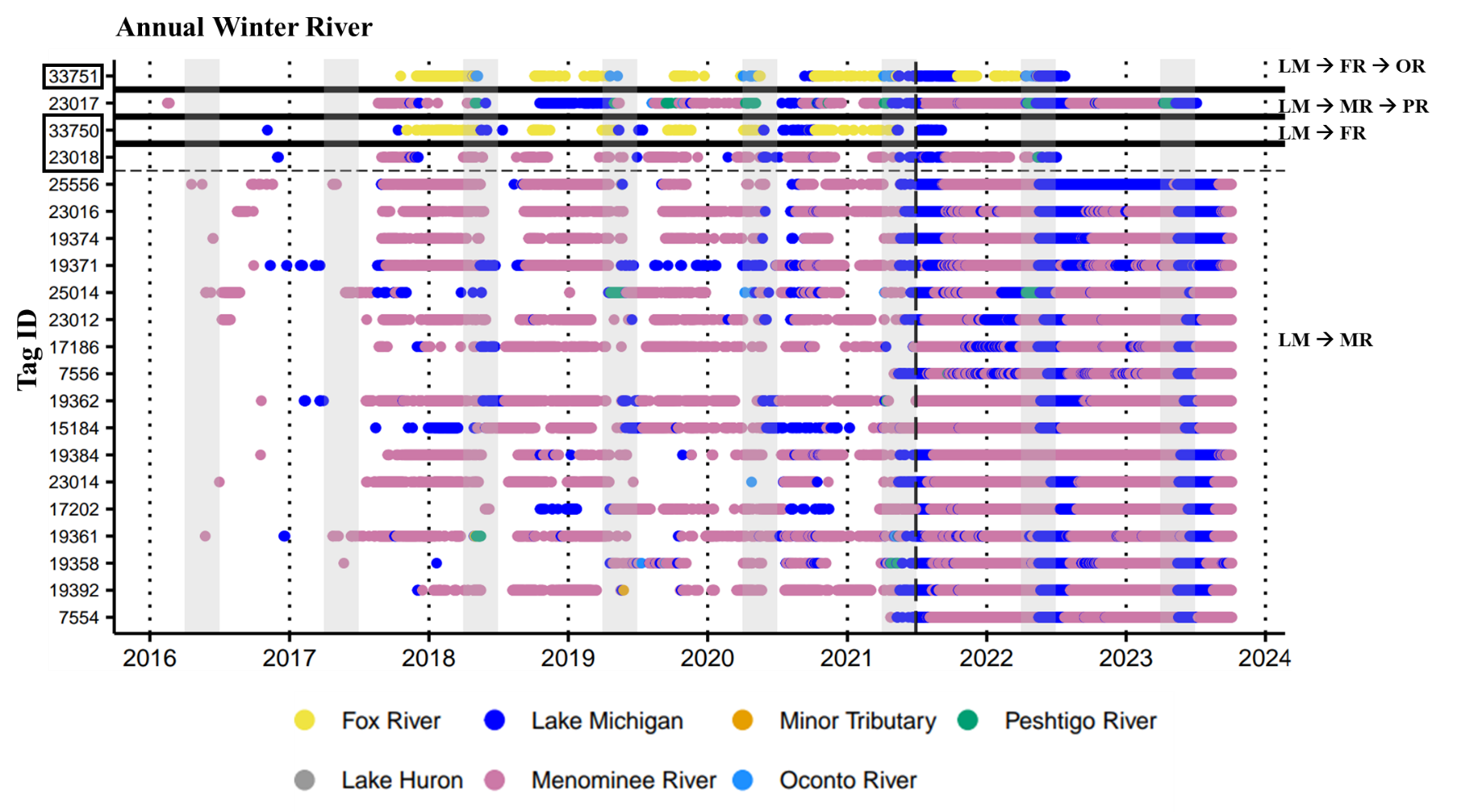


**Supplemental File 7.** Regional sequences displaying the assigned migratory behavior and contingent for all classified individuals (*N*=185) from the Green Bay population. One group of individuals was classified using agglomerative hierarchical clustering followed by visual inspection of habitat and regional sequences (*N*=117) and another group was classified by visual inspection alone (*N*=68). Horizontal dashed lines and tag IDs within black boxes delineate individuals classified by visual inspection alone. Contingents are displayed on the right side of each migratory behavior plot, and all regional names used for contingents are abbreviated: Lake Michigan (LM)**,** Fox River (FR), Menominee River (MR), Oconto River (OR), Peshtigo River (PR). Minor tributaries include the Cedar River, Ford River, Duck Creek, Pensaukee River, Suamico River, Little Suamico River, and Rapid River. Solid horizontal lines delineate contingents, gray bars indicate the typical lake sturgeon spawning season in the Laurentian Great Lakes (April-June), and the vertical dashed line denotes the daily location history time frame used for sequence analyses (6/29/2021 – 10/05/2023). Gaps in sequences prior to the dashed line or for individuals classified via visual inspection alone did not have last observation carried forward applied and therefore display data based solely on receiver detections. Data prior to 2016 were not plotted due to the minimal number of detections present during those years (*N=*117).
