# Supplementary material for: Lake sturgeon behavioral diversity in the Laurentian great lakes: migratory patterns across populations and habitats
Source: Mov Ecol. 2025 Oct 23;13:75. doi: 10.1186/s40462-025-00585-y (PMC12548266; doi:10.1186/s40462-025-00585-y)
Supplement: Supplementary file 8 — Supplementary Material 8 [file 40462_2025_585_MOESM8_ESM.docx]

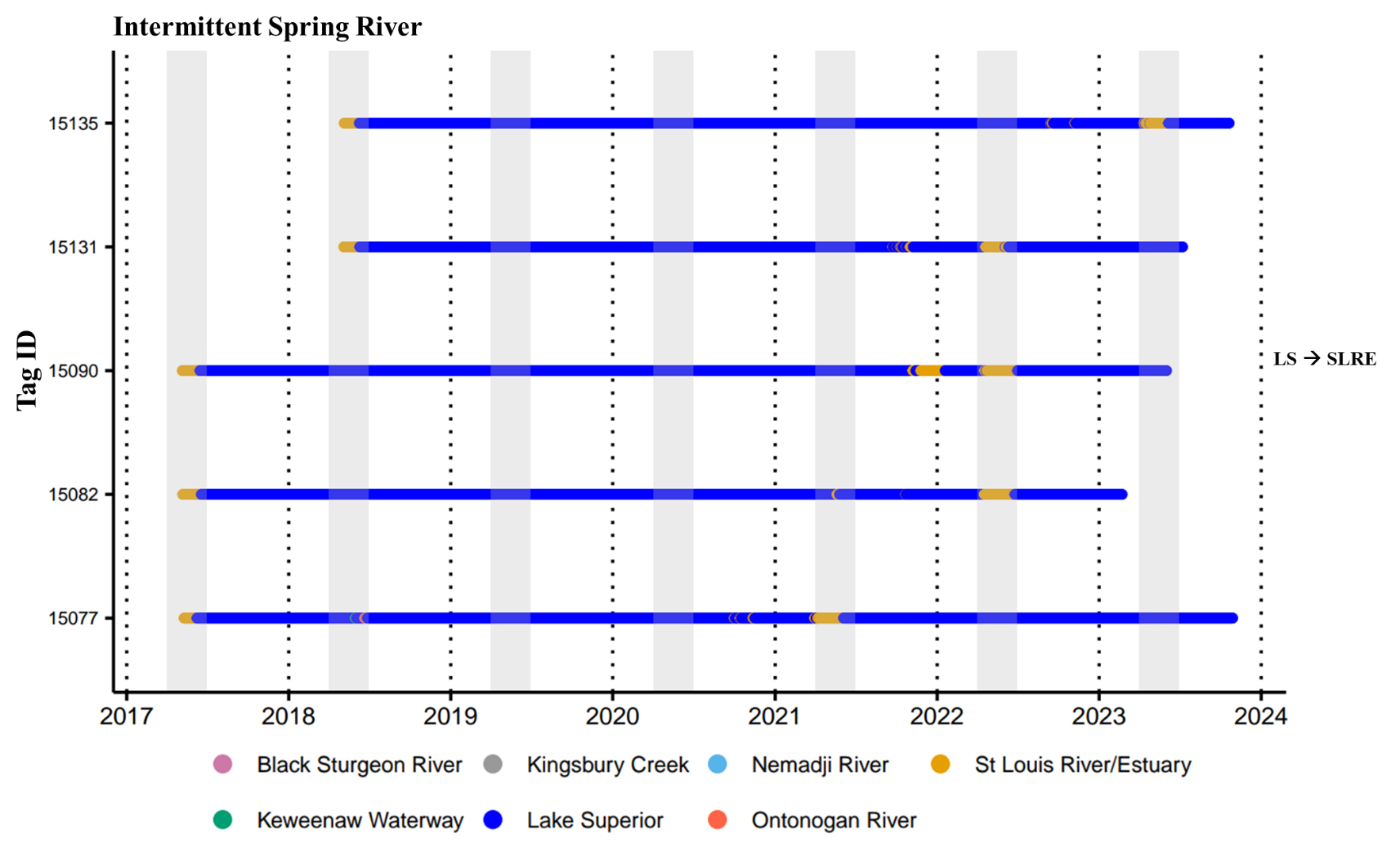


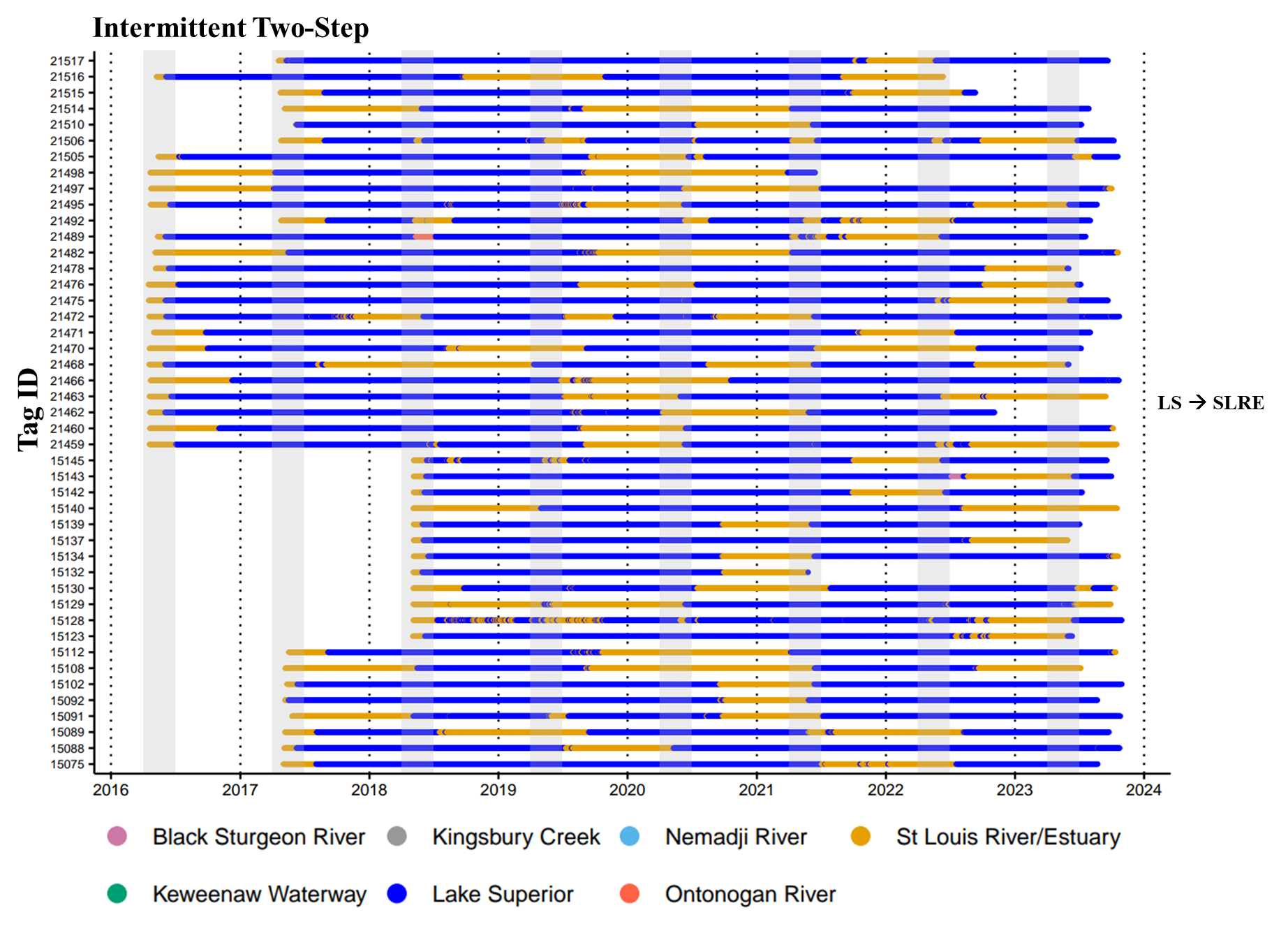


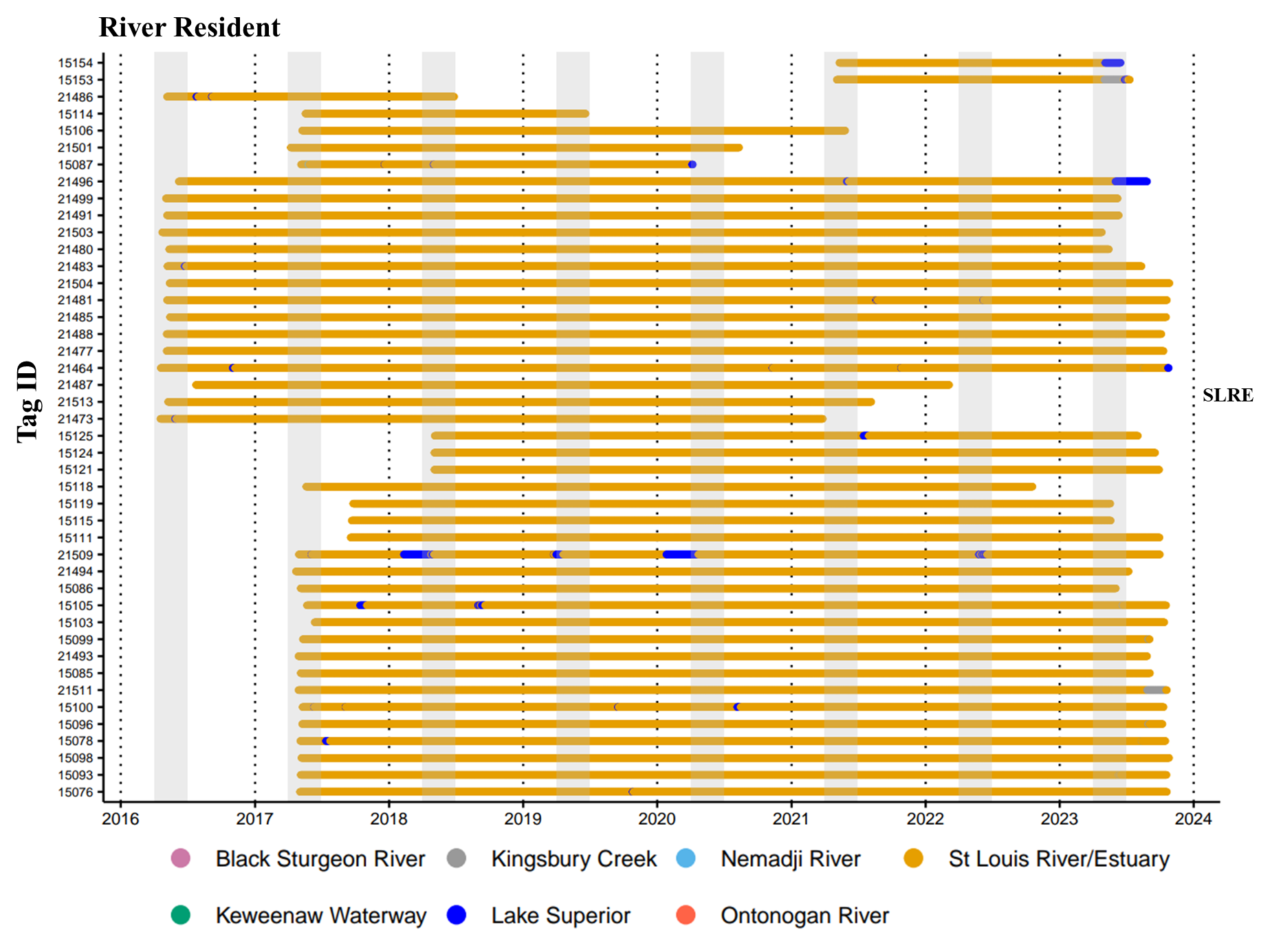


**Supplemental Figure 8.** Regional sequences displaying the assigned migratory behavior and contingent for all classified individuals (*N*=94) from the St. Louis River Estuary population. Individuals were classified using agglomerative hierarchical clustering followed by visual inspection of habitat and regional sequences. Contingents are displayed on the right side of each migratory behavior plot, and all regional names used for contingents are abbreviated: Lake Superior (LS) and St. Louis River Estuary (SLRE).
