# Supplementary material for: Lake sturgeon behavioral diversity in the Laurentian great lakes: migratory patterns across populations and habitats
Source: Mov Ecol. 2025 Oct 23;13:75. doi: 10.1186/s40462-025-00585-y (PMC12548266; doi:10.1186/s40462-025-00585-y)
Supplement: Supplementary file 10 — Supplementary Material 10 [file 40462_2025_585_MOESM10_ESM.docx]

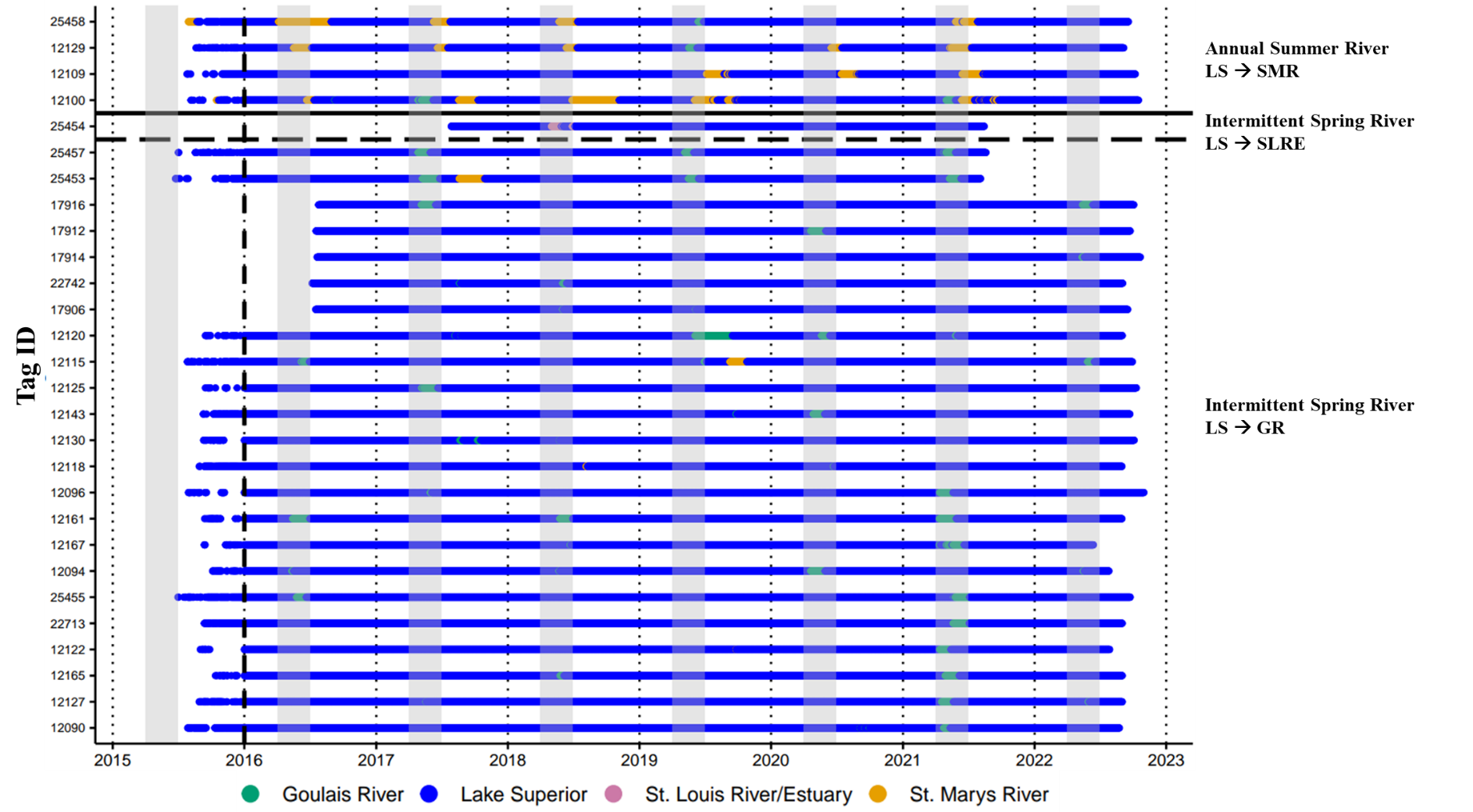


**Supplemental File 10.** Regional behavioral sequences displaying the assigned migratory behavior and contingent for all classified individuals (*N*=28) from the Eastern Lake Superior population. Individuals were classified using agglomerative hierarchical clustering followed by visual inspection of habitat and regional sequences. Migratory behaviors and contingents are displayed on the right side of the plot, and all regional names used for contingents are abbreviated: Lake Superior (LS), Goulais River (GR), St. Marys River (SMR) and St. Louis River Estuary (SLRE). Solid horizontal lines delineate migratory behaviors, dashed horizontal lines delineate contingents, and gray bars indicate the typical lake sturgeon spawning season in the Laurentian Great Lakes (April-June), The vertical dashed line denotes the daily location history time frame used for sequence analyses (1/01/2016 – 10/31/2022). Gaps in sequences prior to and after the dashed line did not have last observation carried forward applied and therefore display data based solely on receiver detections.
