# Supplementary material for: Lake sturgeon behavioral diversity in the Laurentian great lakes: migratory patterns across populations and habitats
Source: Mov Ecol. 2025 Oct 23;13:75. doi: 10.1186/s40462-025-00585-y (PMC12548266; doi:10.1186/s40462-025-00585-y)
Supplement: Supplementary file 11 — Supplementary Material 11 [file 40462_2025_585_MOESM11_ESM.docx]

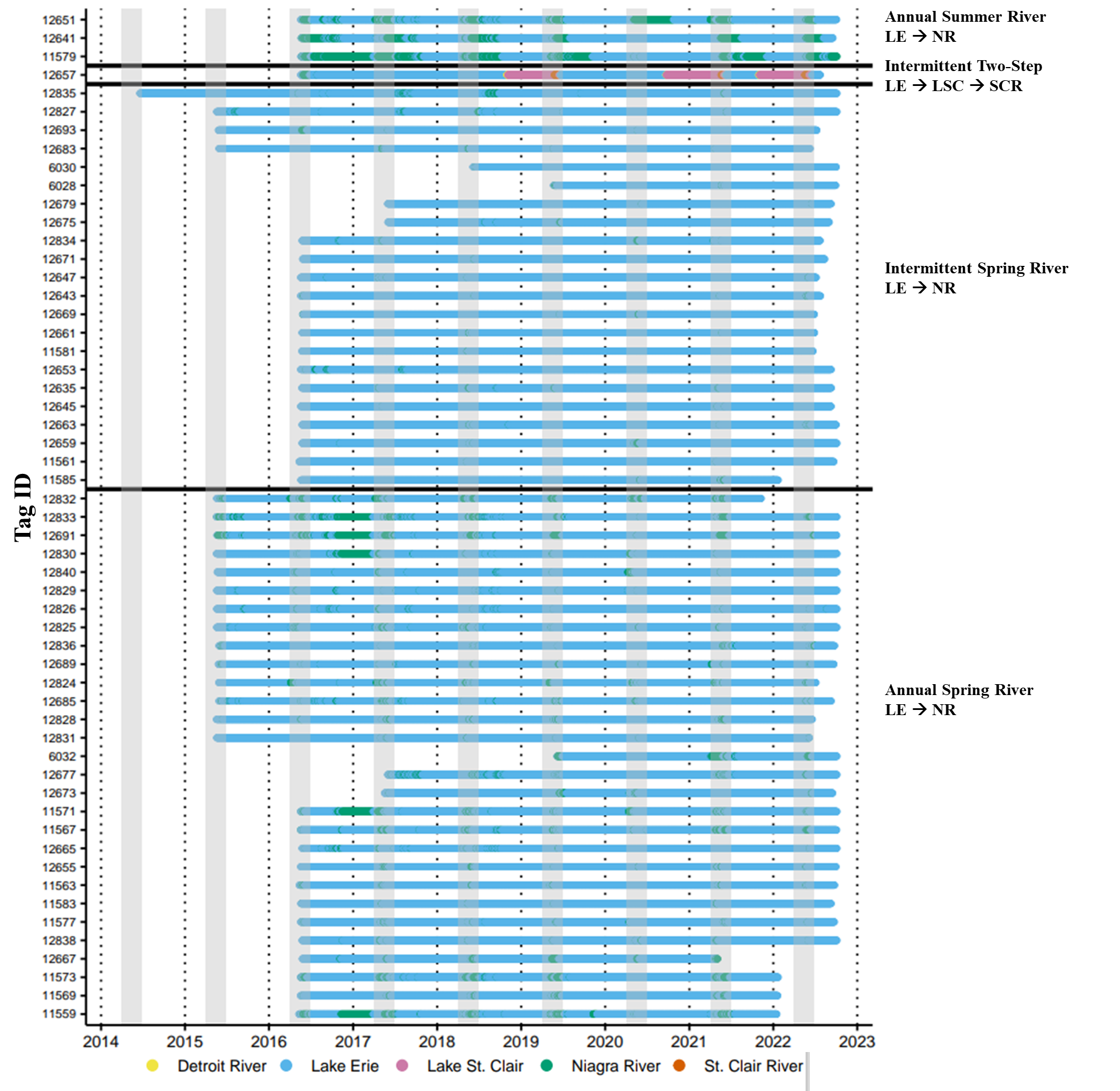


**Supplemental File 11.** Regional behavioral sequences displaying the assigned migratory behavior and contingent for all classified individuals (*N*=55) from the Eastern Lake Erie population. Individuals were classified using agglomerative hierarchical clustering followed by visual inspection of habitat and regional sequences. Migratory behaviors and contingents are displayed on the right side of the plot, and all regional names used for contingents are abbreviated: Lake Erie (LE), Lake St. Clair (LSC), St. Clair River (SCR) and Niagara River (NR). Solid horizontal lines delineate migratory behaviors and gray bars indicate the typical lake sturgeon spawning season in the Laurentian Great Lakes (April-June).
