# Supplementary material for: Lake sturgeon behavioral diversity in the Laurentian great lakes: migratory patterns across populations and habitats
Source: Mov Ecol. 2025 Oct 23;13:75. doi: 10.1186/s40462-025-00585-y (PMC12548266; doi:10.1186/s40462-025-00585-y)
Supplement: Supplementary file 12 — Supplementary Material 12 [file 40462_2025_585_MOESM12_ESM.docx]

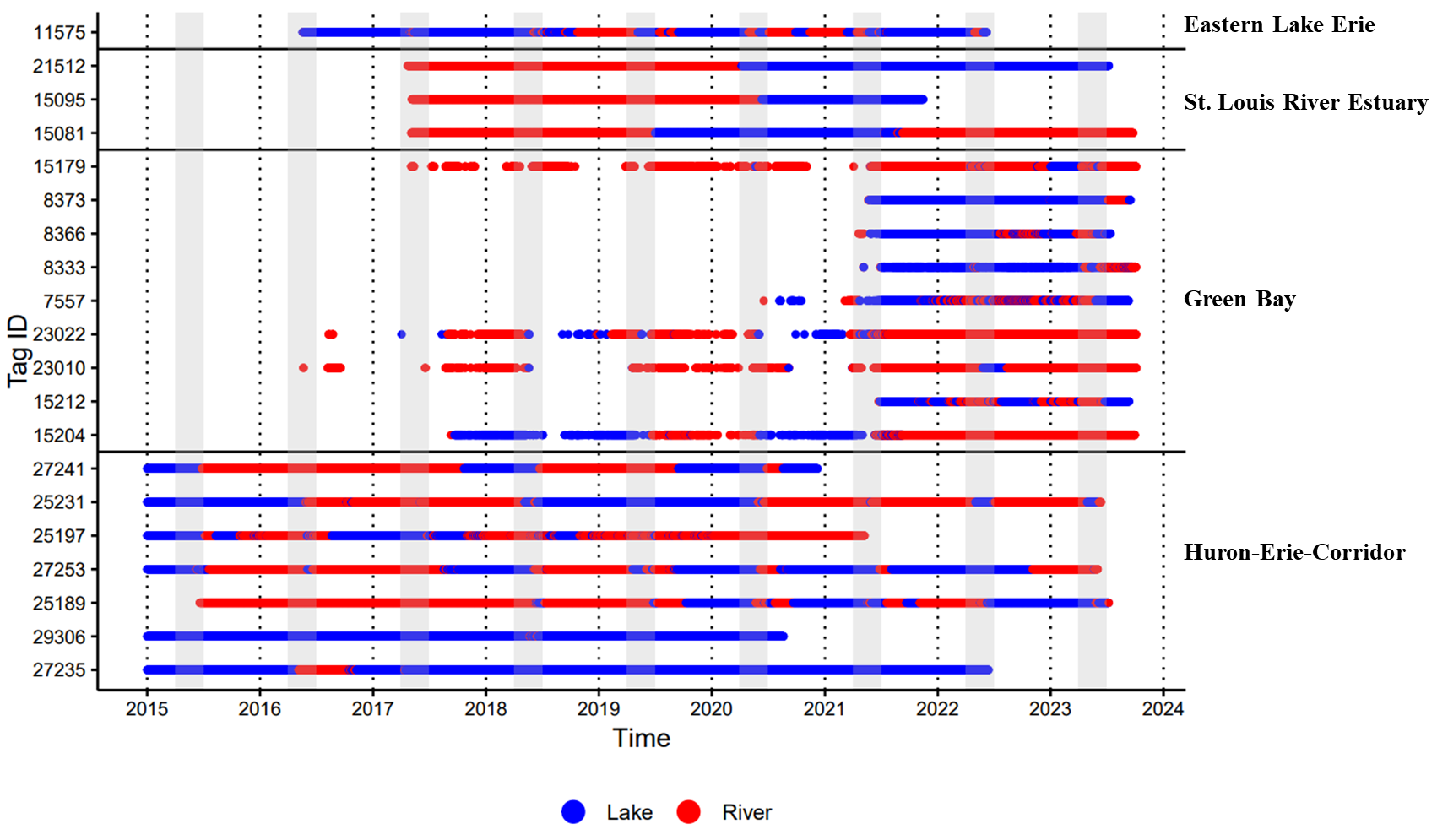


**Supplemental File 12.** Habitat sequences for unclassified lake sturgeon. Individuals were unable to be classified using agglomerative hierarchical clustering and visual inspection due to behavioral sequences not conforming with one of the seven identified patterns of migratory behavior. Solid horizontal lines delineate populations, and gray bars indicate the typical lake sturgeon spawning season in the Laurentian Great Lakes (April-June).
